# Measurement Error Models Enable Accurate Correlation of Nanoscale Properties and Structures

**DOI:** 10.1021/acsnano.5c12141

**Published:** 2026-08-06

**Authors:** Adam L. Pintar, Andrew C. Madison, Craig R. Copeland, Natalia Farkas, Samuel M. Stavis

**Affiliations:** † Statistical Engineering Division, 10833 National Institute of Standards and Technology, Gaithersburg, Maryland 20899, United States; ‡ Microsystems and Nanotechnology Division, National Institute of Standards and Technology, Gaithersburg, Maryland 20899, United States; § Theiss Research, La Jolla, California 92037, United States; ∥ Building and Fire Sciences, United States Forest Service, Forest Products Laboratory, Madison, Wisconsin 53726, United States

**Keywords:** statistical, variance, structure−property, microscopy, fluorescence, scattering

## Abstract

Measurement errors can catastrophically bias parameter estimates in models to correlate nanoscale properties and structures, undercutting a foundation of nanoscience and nanotechnology. In a general solution to this latent problem, we derive a method-of-moments correction for least-squares estimates of correlative model parameters. Our correction applies to many relations of dependent and independent variables, subject to many types of measurement errors of the independent variable. Focusing on the prevalent power-law model to correlate optical intensity and nanoparticle size, we test the limits of accuracy of our correction and study the use of either reference size distributions or sizing uncertainty estimates to inform a measurement error model. We critically evaluate representative measurements of various nanoparticles, impacting the conclusions of several studies and changing expectations of intensity scaling exponents for nanoscale optical inferences. Our correction is immediately available, highly interpretable, and broadly applicable to gain reliable insights and prevent future mistakes.

## Graphical abstract

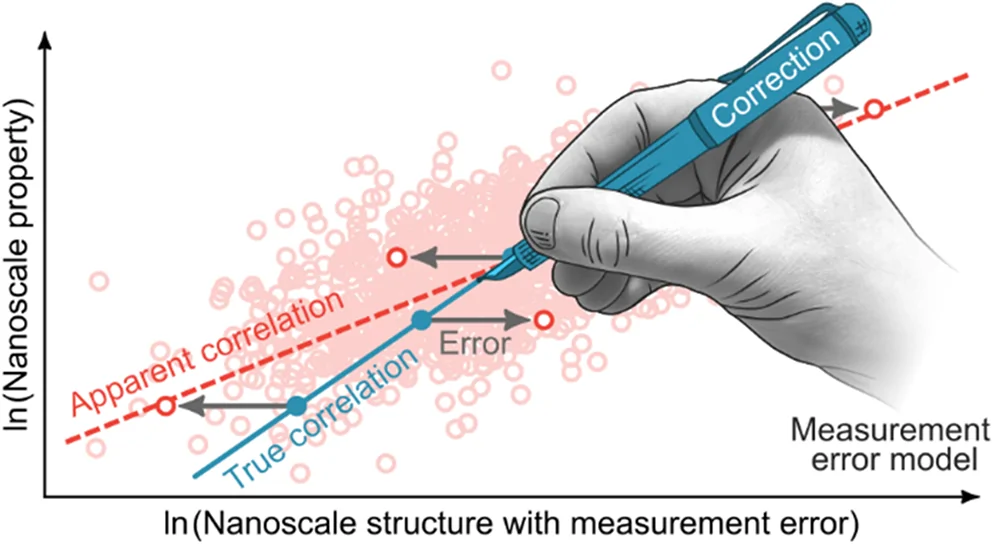

Correlation of nanoscale properties and structures is a foundation of nanoscience and nanotechnology. Accordingly, estimation of parameter values of correlative models is important for nanoparticles, which manifest property distributions that depend on size distributions.^1^ For example, semiconductor nanocrystals are luminescent in consumer electronics,^2^ while perovskite nanostructures absorb light efficiently in photovoltaic cells.^3^ Metal nanoclusters and nanoparticles are reactive in catalysts and antimicrobials,^4^ whereas ceramic nanoparticles are stable for medical implants.^5^ Lipid vesicles carry pharmaceuticals to prevent and treat disease,^6^ yet nanoplastic particles sorb chemicals and present potential hazards.^7^ In each example, property–size correlation is essential to understand the fundamental nanoscience, engineer a practical nanotechnology, or evaluate any potential nanotoxicity. Yet, methods to analyze such correlation contend with the ubiquitous but underappreciated effects of sizing errors.^8–15^

Presently, we study how measurement errors of the independent variable, such as sizing errors, can bias parameter estimates in correlative models. While we address this critical issue from a general perspective, we focus on a correlative model of particular interest, the ordinary power law, *y* = α*x* ^β^, with dependent variable, *y*, coefficient, α, independent variable, *x*, and exponent, β. This model is prevalent in the literature,^8–15^ as the exponent models the scaling dependence of a physical,^16^ chemical,^17^ interfacial^18^ or other property on the size of a nanoparticle. Among these scaling dependences, optical properties such as light scattering intensity and fluorescence emission intensity are interesting in their own right and are also indicators of other interesting properties, such as refractive index and molecular loading. Moreover, fluorescence emission is useful to detect polymeric nanoparticles that often serve as size standards^19^ to test sizing methods.^10–15^ Such nanoparticles might also serve as intensity standards to support efforts to use expected exponents to interpret^15^ and even validate^9,13^ measurement results, or to infer particle size from fluorescence intensity by fitting and inverting a power-law model.^20,21^ In the presence of sizing errors, however, intensity–size correlation becomes problematic for these and other applications.^22^

Correlative analyses often involve transformations of measurement data to linearize trends and simplify parameter estimation. Logarithmic transformation of a power-law trend to yield a linear trend is common, and bias of the corresponding slope estimate due to measurement errors of the independent variable is a classic problem in statistics. Yet, custom solutions are necessary to meet present demands. Several statistics textbooks^23–25^ consider measurement errors in a range of situations, ranging from fitting a line to two variables, to generalized linear models.^26^ Such analyses have improved accuracy in fields of study such as econometrics^27^ and force calibration.^28^ Even so, ignoring significant errors of the independent variable is a common mistake that conflates apparent and true values. The best practice is to characterize an apparent value as a function of the true value and a random variable modeling measurement error. This function is a measurement error model, which can greatly improve the accuracy of parameter estimation relative to ordinary least-squares regression.

Following a related exchange in *ACS Nano*,^12,13,22^ we delve deeper into this latent and likely pervasive problem. In the presence of sizing errors, observation of an expected exponent, which would ordinarily build confidence, could instead result from suppression or attenuation of an unexpected exponent. Seemingly small sizing errors can cause surprisingly large biases,^22^ yielding misleading insights. To address this issue, we derive, test, and apply a general method-of-moments correction of such biases. Our analytic expression is approximate, with limits of accuracy, whereas ignoring sizing errors tends to incur a biased exponent. Our correction is immediately available, highly interpretable, and broadly applicable to gain reliable insights and prevent future mistakes.

Our approach toward this correction differs from standard approaches^23–25^ in three ways. First, we allow for a bias of mean size, which interacts unexpectedly with any bias of the standard deviation of the particle size distribution, commonly an excess variance.^22^ Second, we consider a general model of sizing errors, capable of capturing various random and systematic effects in experimental measurements. Third, with different simplifications, our corrections for additive and multiplicative errors converge to the same compact approximation in terms of overt measurement parameters rather than latent error parameters, allowing for direct use of sizing results as correction inputs.

Either reference data for the independent variable or an uncertainty estimate from a measurement function^29^ can inform a measurement error model. A caveat is that the accuracy of the model and the resulting correction depends on the reliability of the underlying information.^23^ Ideally, reference data are available, such as the size distribution of a nanoparticle standard.^19^ Otherwise, an uncertainty evaluation using a measurement function can suffice, although the standard approach assumes the correction of bias^30^ and often leads to an underestimate of uncertainty.^31^ In this context, we introduce a guide to determining measurement errors and correcting least-squares estimates of power-law exponents (Figure 1), demonstrating its use and testing its limits in our subsequent evaluation of several studies.

**Figure 1:**
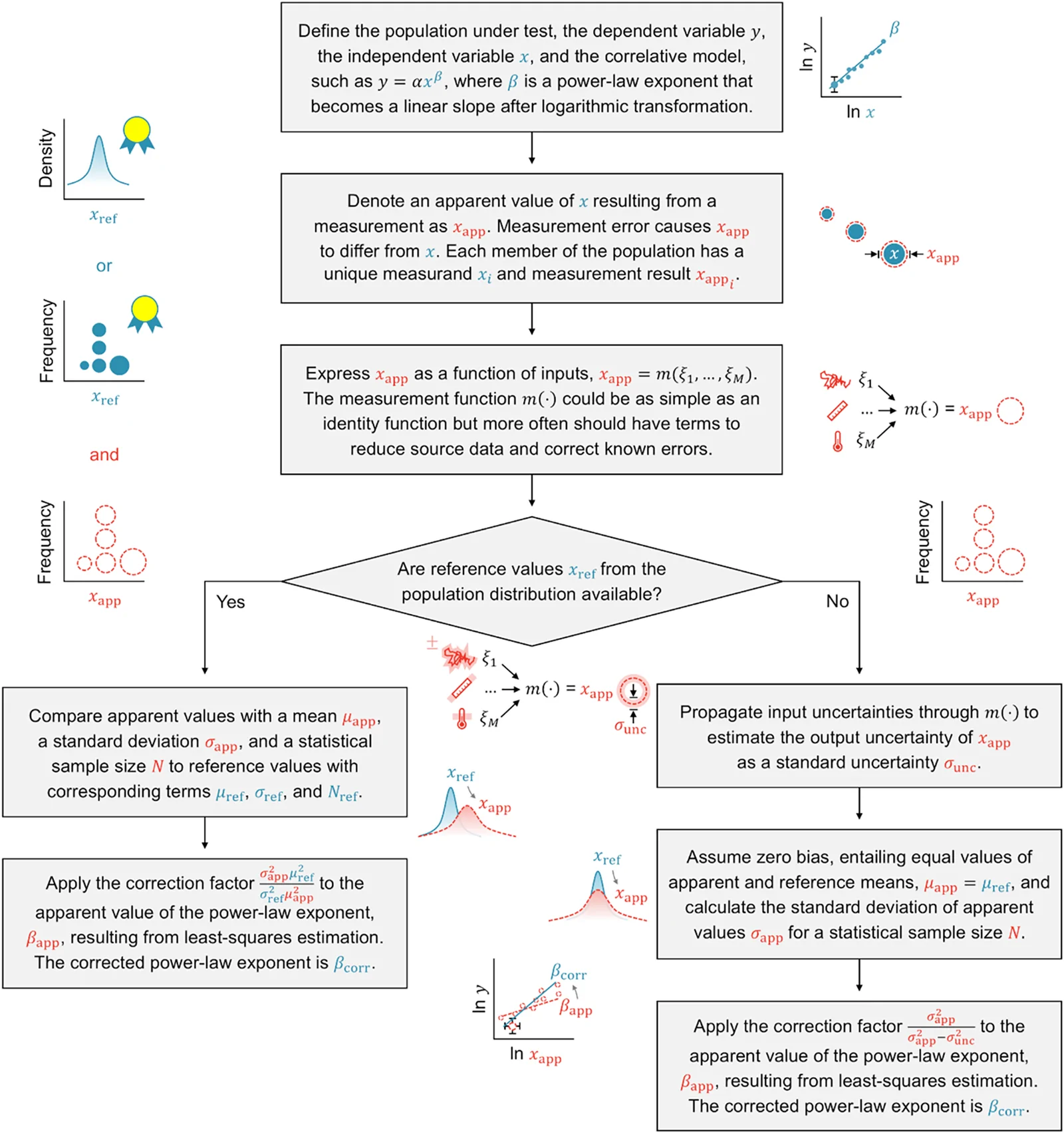
Correction Guide. This guide summarizes how to determine measurement errors of an independent variable and correct a power-law exponent that models a dependent variable. The terms *x* and *y* are generic and can represent different independent and dependent variables, respectively. The left branch of the guide involves determining measurement errors from reference data. The right branch of the guide involves determining measurement errors from uncertainty estimates.

Our guide is statistically formal but still useful to nonstatisticians, with the following steps to improve correlative accuracy. First, define the dependent and independent variables, and the power law or another correlative model (Table S1 and Note S1). Next, consider the measurement process that yields the independent variable, expressing a measurement function that relates the input of source data to the output of a measurement result (Note S2). Such an expression helps to identify and correct bias, and allows for a formal estimate of measurement uncertainty.

Then, at the branch point of our guide, consider the availability of reference data for the independent variable. If reference data are available, determine the mean and standard deviation of the apparent values and of the reference data, and input those four values to obtain a correction factor for the least-squares estimate of the power-law exponent. If reference data are unavailable, use the uncertainty estimate from the measurement function (Note S2), along with the standard deviation of the apparent values of the independent variable, to determine a correction factor.

## Results and Discussion

### Measurement Error Types

To accurately correlate a dependent variable with the independent variable of particle size, any valid statistical analysis must account for sizing errors. To do so, measurement error models map apparent sizes to true sizes, or reference sizes in an experimental approximation of the truth, by a mathematical function. Our correction considers functions of the form *x*_app_ = *h*(*x*, ϵ) ≈ *h*(*x*_ref_, ϵ), where *x*_app_ is the apparent radius, *x* is the true radius, ϵ is a random variable modeling error in a measurement process, and *x*_ref_ is a reference radius. Theoretically, we differentiate between *x* and *x*_ref_. Practically, we assume that *x*_ref_ is equivalent to *x*, revisiting the limitations of this assumption. In measurement processes, two common forms of error are additive, *x*_app_ = *x*_ref_ + ϵ_a_, and multiplicative, *x*_app_ = ϵ_m_ *x*_ref_, where ϵ_a_ and ϵ_m_ are random variables with distributions, normal by default, modeling additive and multiplicative errors. Particle radii are natural to consider as the independent variable in scaling relationships and our subsequent analysis. If the value of radius is unimportant, then we discuss particle size using the generic term.

The additive and multiplicative models can capture both random and systematic errors of the independent variable. The additive model approximates offset errors, such as incorrect determination of an edge threshold in the analysis of nanoparticle images from electron microscopy, or divergent measurands of hydrodynamic size from tracking analysis and steric size from electron microscopy. The multiplicative model approximates scaling errors, such as those due to magnification miscalibration in electron or optical microscopy, or inaccurate thermometry, viscometry, or hydrodynamic hindrance correction in tracking analysis.^22^ Either model could account for errors that result from the reduction of complex shapes to equivalent sizes by electron microscopy. All of these errors, and errors of other types, could be present in the measurement results that we subsequently evaluate.

### Correlative Model Corrections

We derive a correction for least-squares estimates of correlative model parameters of the form *f*(*y*) = α + β*g*(*x*_ref_) + δ, where *f*(·) and *g*(·) are continuous functions, δ is a random variable denoting an additive error, and *x*_app_ = *h*(*x*_ref_, ϵ) is a measurement error model (Table S1 and Note S1). The generality of our correction makes it broadly applicable, such as to some polynomial and exponential models. Two corrections of particular interest, which we term PLE+ or PLE×, apply to a power-law exponent subject to additive or multiplicative errors, respectively. To relate the ordinary power law to our general correlative model, we recast it as *y* = δ_trans_α_trans_ *x*_ref_^β^ such that *f*(*y*) = ln(*y*), *g*(*x*_ref_) = ln(*x*_ref_), δ = ln(δ_trans_), α = ln(α_trans_), and *h*(*x*_ref_, ϵ_a_) = *x*_ref_ + ϵ_a_ or *h*(*x*_ref_, ϵ_m_) = ϵ_m_ *x*_ref_. We note that logarithmic transformations of measurement results with explicit units require division of the results by a reference value to achieve dimensionless ratios (Note S1).

We express the corrections of the exponent, β, in terms of the mean and standard deviation of the reference radius distribution, μ_ref_ and σ_ref_, and the mean and standard deviation of the latent error, either μ_a_ and σ_a_ for additive error, ϵ_a_, or μ_m_ and σ_m_ for multiplicative error, ϵ_m_. Upon availability of overt measurement results for the mean and standard deviation of the apparent radius distribution, μ_app_ and σ_app_, PLE+ and PLE× converge to a compact approximation, which we term PLE≈. Each of these three correction factors scales an apparent exponent, β_app_, that results from least-squares estimation to a corrected exponent

1 $$\beta_{\mathrm{corr}}=\beta_{\mathrm{app}}\left[\frac{\sigma_{\mathrm{ref}}^{2}+\sigma_{a}^{2}}{\sigma_{\mathrm{ref}}^{2}}\right]{\left[1+\frac{\mu_{a}}{\mu_{\mathrm{ref}}}\right]}^{-2}$$

for additive errors,

2 $$\beta_{\mathrm{corr}}=\beta_{\mathrm{app}}\left[\frac{\frac{\sigma_{\mathrm{ref}}^{2}}{\mu_{\mathrm{ref}}^{2}}+\frac{\sigma_{m}^{2}}{\mu_{m}^{2}}}{\frac{\sigma_{\mathrm{ref}}^{2}}{\mu_{\mathrm{ref}}^{2}}}\right]$$

for multiplicative errors, and

3 $$\beta_{\mathrm{corr}}\approx \beta_{\mathrm{app}}\left[\frac{\sigma_{\mathrm{app}}^{2}\mu_{\mathrm{ref}}^{2}}{\sigma_{\mathrm{ref}}^{2}\mu_{\mathrm{app}}^{2}}\right]$$

for the compact approximation. The PLE≈ correction is simple, avoiding both a choice between the PLE+ and PLE× corrections and a calculation of latent error parameters. Figures 2a–c show maps of eqs 1–3 as functions of the relative error of the mean, ϵ_μ_ μ_ref_^–1^ = (μ_app_ – μ_ref_)­μ_ref_^–1^, and the relative error of the standard deviation, ϵ_σ_σ_ref_^–1^ = (σ_app_ – σ_ref_)­σ_ref_^–1^ for a representative radius distribution with μ_ref_ = 25 nm and σ_ref_ = 2.5 nm. In plots of correction factors, white is unity, shades of crimson are above unity, and shades of gray are below unity. These equations involve comparisons of apparent and reference size distributions, but the latter may be unavailable, which we revisit. Importantly, the three correction factors are independent of both the apparent and true values of the exponent, but some subtle differences between the corrections are still evident, as follows.

**Figure 2:**
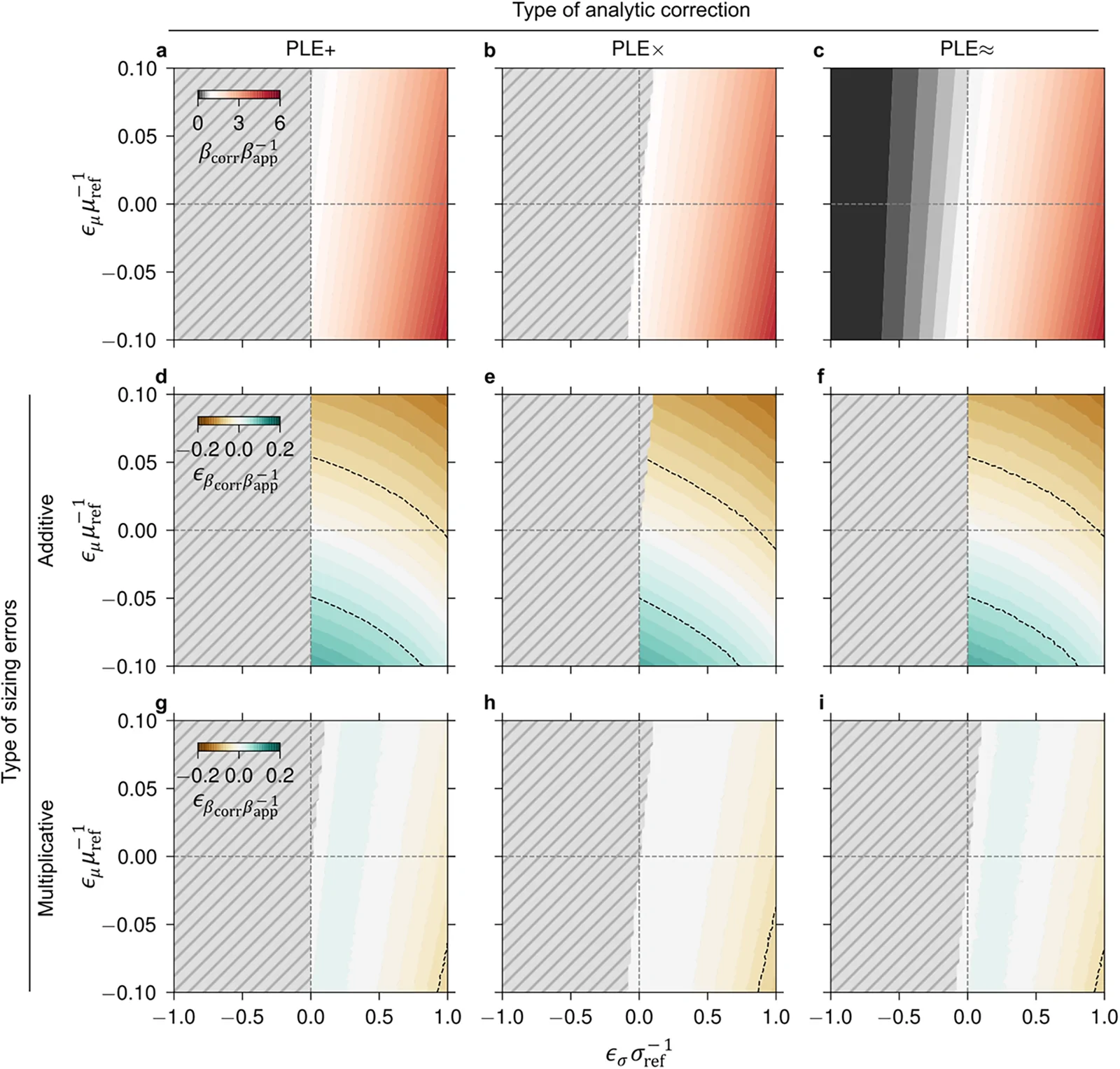
Correction Summary. (a–c) Contour plots showing correction factors from three types of corrections for least-squares estimates of power-law exponents. (d–i) Contour plots showing a matrix of relative errors of the correction factors for (d–f) additive and (g–i) multiplicative sizing errors. The plots show (a, d, g) PLE+, (b, e, h) PLE×, and (c, f, i) PLE≈ corrections as functions of the relative error of the standard deviation, ϵ_σ_σ_ref_^–1^, and the relative error of the mean, ϵ_μ_μ_ref_^–1^ for a reference radius distribution with μ_ref_ = 25 nm and σ_ref_ = 2.5 nm. Gray hatch regions are invalid parameter combinations. Black dash contours in (d–i) bound a relative error of the correction factors of ±5%.

The PLE+ correction (Figure 2a) requires ϵ_σ_σ_ref_^–1^ to be positive, such that the apparent size distribution is always broader than the reference size distribution due to random effects that increase uncertainty. In contrast, ϵ_μ_μ_ref_^‑1^ can be either positive or negative. The correction increases with ϵ_σ_σ_ref_^–1^. Nonintuitively, for a particular ϵ_σ_σ_ref_^–1^, the correction becomes less severe as ϵ_μ_μ_ref_^–1^ increases. This compensation balances on the line ϵ_σ_σ_ref_^–1^ = ϵ_μ_μ_ref_^–1^ (white region of Figure 2a), where errors of the mean and standard deviation cancel, negating any bias of the power-law exponent.

We previously observed and can now explain this interesting cancellation of particular errors^22^ as a balance of opposing effects. For ϵ_σ_σ_ref_^–1^ > 0, positive errors of the standard deviation broaden the distribution of *x*_app_ relative to the distribution of *x*_ref_, attenuating the apparent power-law exponent by expanding the range of the independent variable. For ϵ_μ_μ_ref_^–1^ > 0, however, positive errors of the mean shift the distribution of *x*_app_ to larger values relative to the distribution of *x*_ref_. The nonlinear compressive effect of the logarithmic transformation on *x*_app_ is greater than for *x*_ref_, enhancing the apparent power-law exponent. This condition is a specific property of the logarithmic transformation of the independent variable rather than a general property of measurement error models.

The PLE× correction (Figure 2b) is similar to the PLE+ correction, except that ϵ_σ_σ_ref_^–1^ can be negative. Positive and negative values of ϵ_σ_σ_ref_^–1^ correspond, respectively, to a broader and narrower distribution of *x*_app_ relative to the distribution of *x*_ref_ from random or systematic effects that scale *x*_ref_. The restriction on PLE× is (ϵ_σ_σ_ref_^–1^ + 1)^2^ ≥ (ϵ_μ_μ_ref_^–1^ + 1)^2^. For the condition of equality, the correction factor is unity. The PLE≈ correction (Figure 2c) is identical to the PLE+ correction for ϵ_σ_σ_ref_^–1^ > 0, since eq 1 and eq 3 are algebraic manipulations of one another. However, eq 3 imposes no restrictions on ϵ_σ_σ_ref_^–1^ or ϵ_μ_μ_ref_^–1^ and so applies to any combination thereof. The correction predicts that negative values of ϵ_σ_σ_ref_^–1^ enhance, rather than attenuate, least-squares estimates of power-law exponents, with the corresponding positive bias increasing as ϵ_σ_σ_ref_^–1^ decreases. Even though eq 3 yields a correction for any combination of ϵ_σ_σ_ref_^–1^ and ϵ_μ_μ_ref_^–1^, caution is appropriate if neither the additive nor the multiplicative measurement error models apply, as for ϵ_σ_σ_ref_^–1^ < 0 and (ϵ_σ_σ_ref_^–1^ + 1)^2^ < (ϵ_μ_μ_ref_^–1^ + 1)^2^. For completeness, we show maps of eq 3 for all coordinates in Figure 2c.

Despite these subtle differences among eqs 1–3, the three models yield similar corrections for ϵ_σ_σ_ref_^–1^ > 0, which is a common issue in particle sizing.^10,13–15^ The PLE+ and PLE≈ corrections are identical and the PLE× and PLE≈ corrections differ by less than 1% due to a simplifying approximation relating the latent and overt measurement parameters for PLE× (Note S1). This agreement shows that fundamentally different forms of error of the independent variable can have similar effects on exponent bias, leading to similar corrections.

A tutorial example applies the PLE≈ correction to synthetic data, demonstrating the calculation for small statistical samples of populations under test (Note S3 and Table S2). This example also shows the resulting potential for large errors of correction factors due to sampling variability, which could be high for low counts of single particles resulting from microscopy measurements with low throughput.

### Correction Accuracy Tests

We test the accuracy of eqs 1–3 by simulations in which true distributions of sizing errors corrupt true intensity–size correlations to yield erroneous exponents, which we correct for comparison to the true values from the initial correlation (Figures 2d–i, 3, and S1–S9). The results show that the accuracy of a correction depends on the true measurement error model and the values of ϵ_σ_σ_ref_^–1^, ϵ_μ_μ_ref_^–1^, σ_ref_, and μ_ref_. Figures 2d–i show test results for exemplary true values of β = 6 and a reference radius distribution with μ_ref_ = 25 nm and σ_ref_ = 2.5 nm. We pair each true measurement error model, either PLE+ or PLE×, with every available correction, either PLE+, PLE×, or PLE≈. We evaluate these combinations across many values of ϵ_σ_σ_ref_^–1^ and ϵ_μ_μ_ref_^–1^. In plots of relative errors of correction factors, white is zero, shades of green are above zero, and shades of ochre are below zero. The correction factors in eqs 1–3 are accurate for combinations of ϵ_σ_σ_ref_^–1^ and ϵ_μ_μ_ref_^–1^ that are white, or nearly so, in Figure 2d–i. Relative errors of correction factors of ±5% appear as black dash contours in Figure 2d–i.

**Figure 3:**
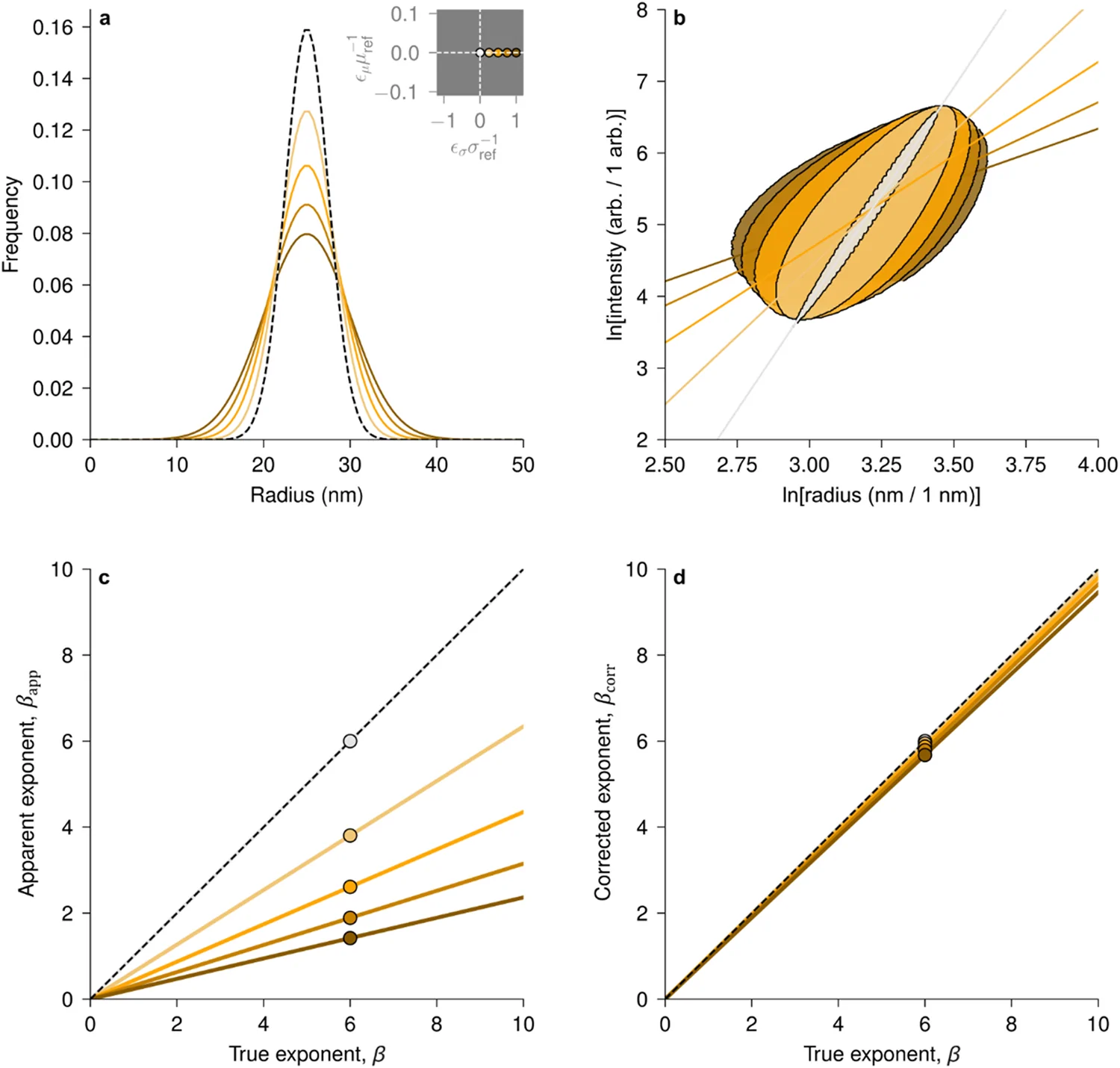
Correction Accuracy. This figure shows the scenario of additive errors of zero mean and varying standard deviations. Figures S1–S3 show other relevant scenarios. (a) Probability densities showing (black dash) reference radius distribution with μ_ref_ = 25 nm and σ_ref_ = 2.5 nm subject to (shades of orange) additive errors of ϵ_μ_μ_ref_^–1^ = 0 and ϵ_σ_σ_ref_^–1^ ranging from 0% to 100%. (a, inset) Map showing relative errors of the standard deviation, ϵ_σ_σ_ref_^–1^, and relative errors of the mean, ϵ_μ_μ_ref_^–1^, with respect to the reference values. (b) Plots showing joint histograms of ln [intensity (arb./1 arb.)] versus apparent ln [radius (nm/1 nm)] for a true exponent of β = 6, subject to the additive errors of radius in (a) and additive errors of ln [intensity (arb./1 arb.)] of zero mean and a standard deviation of 0.05 ln (arb./1 arb.). Solid lines result from least-squares regression. Elliptical and oblong regions cover 95% of these errors. (c, d) Plots showing (c) apparent exponents and (d) corrected exponents as a function of the true exponent for the relative errors of the standard deviation in (a). Circles in (c) and (d) correspond to a true exponent of β = 6. Line thicknesses in (c) and (d) cover 68% of statistical sampling variability. The black dash lines in (c) and (d) indicate an equality of true and apparent or corrected exponents.

An accurate correction is possible for many true values of the exponent (Figure 3). We test the performance of the PLE+ true model and correction for 0 ≤ β ≤ 10, values of ϵ_σ_σ_ref_^–1^ up to 100%, ϵ_μ_μ_ref_^–1^ = 0, and a reference radius distribution with μ_ref_ = 25 nm and σ_ref_ = 2.5 nm. As ϵ_σ_σ_ref_^–1^ increases, the apparent size distribution broadens (Figure 3a) and the exponent attenuation deepens (Figure 3b,c), but eq 1 still yields an accurate correction to within a relative error of ±6% (Figure 3d) when β = 6. Different combinations of ϵ_σ_σ_ref_^–1^, ϵ_μ_μ_ref_^–1^, PLE+, and PLE× yield similar results (Figures S1–S3).

Correction accuracy evidently depends on the parameters σ_ref_ and μ_ref_ through their ratio σ_ref_μ_ref_^–1^, which is the coefficient of variation of the particle size distribution. Two comparisons show this dependence. First, in a comparison of Figure 2d–i, with μ_ref_ = 25 nm and σ_ref_ = 2.5 nm, to Figure S4d–i, with μ_ref_ = 50 nm and σ_ref_ = 5.0 nm, the correction accuracy is indistinguishable for the same coefficient of variation of σ_ref_μ_ref_^–1^ = 0.1. In a second comparison of Figure 2d–i to S6d–i, with μ_ref_ = 18.5 nm, σ_ref_ = 3.1 nm, and σ_ref_μ_ref_^–1^ = 0.17, the correction accuracy worsens as the coefficient of variation increases.

This degradation occurs in part because our derivation involves the standard approximation for Var­[ln­(*x*_ref_)] as σ_ref_^2^μ_ref_^–2^, which breaks down as σ_ref_μ_ref_^–1^ increases. The approximation error admits an analytic solution for a log-normal distribution of *x*_ref_ and occurs generally. Therefore, while our correction is usefully accurate for many distributions of nanoparticle size, samples with high coefficients of variation incur the expectation of some residual correlative bias. Improving correlative accuracy for broader size distributions motivates further development of our methodology, such as applying better approximations or adopting exact inference procedures that require numerical solutions.

Supporting simulations test the sensitivity of correction accuracy to either normal or log-normal distributions of reference size, which are both common approximations of particle size distributions. We find only a small dependence on the selection of either normal or log-normal distributions of reference size on exponent attenuation for each error model (Table S3). Because exponent attenuation is insensitive to either normal or log-normal distributions of reference size, corrections that are accurate for one distribution will also be accurate for the other.

Modifications of eqs 1 and 2 could improve accuracy at the cost of complexity (Note S1), should that trade-off be desirable. A modification of eq 1 accounts for potentially non-negligible correlation of the two terms of a linear approximation but precludes the compact approximation. A modification of eq 2 improves the accuracy of the expression for Var­[*x*_app_], and the compact approximation remains valid.

### Literature Evaluation

The main idea of this study is that measurement errors of the independent variable can bias least-squares estimates of correlative model parameters, requiring corrections to improve accuracy. To begin to understand the impact of this idea in the context of correlating nanoscale properties and structures, we critically evaluate several representative studies of nanoparticles in which accounting for sizing errors can affect property–structure correlation and even change conclusions. We divide our literature corrections into three categories. The first category includes studies that report reference size distributions,^10,13–15^ enabling nominally accurate corrections, although large sizing errors degrade correction accuracy. The second category includes studies that report estimates of sizing uncertainty,^8,9^ enabling probable undercorrections that are likely better than the alternative of ignoring sizing errors. These first and second categories correspond respectively to the lower-left and lower-right branches of our guide (Figure 1). We summarize the results from these two categories in Table 1, providing further detail in Table S4, considering only results that are available within the publications, and noting issues that arise in evaluating nonuniform reports. The third category includes studies that report neither reference size distributions nor sizing uncertainty estimates,^20,21^ disallowing any correction but still meriting qualitative evaluation and discussion.

**Table 1:**
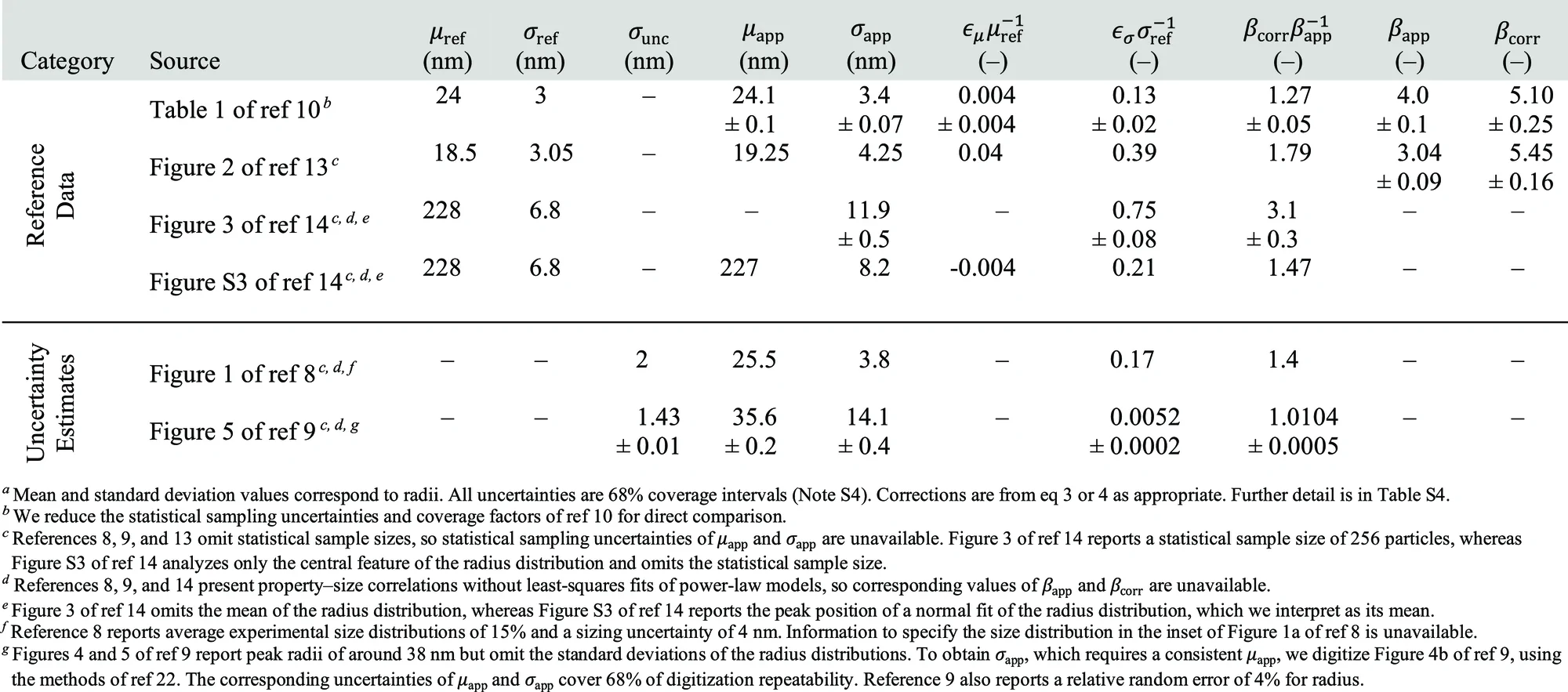
Literature Evaluation^***a***^

### Reference Data

If a reference size distribution is available for comparison to an apparent size distribution, then determining a correction simply involves eq 3, which we apply to the following studies.

In one study of polystyrene nanoparticles,^10^ a supervolumetric scaling of fluorescence intensity was apparent even before accounting for attenuation of the power-law exponent. This study combined nanofluidic size exclusion and localization optical microscopy to measure steric sizes and fluorescence intensities, estimating an apparent exponent of 4.0 ± 0.1 (Table 1 of ref 10). A comparison of apparent and reference sizes indicated relative errors of the mean of 0.4% and of the standard deviation of 13%. Equation 3 corrects the exponent to 5.10 ± 0.25, maintaining the conclusion of supervolumetric scaling but significantly increasing the estimate of the exponent. Simulations indicate good accuracy for this correction (Figure S5).

Another study reported a volumetric scaling of fluorescence intensity for similar nanoparticles.^13^ This study applied nanoparticle tracking analysis in confining microwells to measure hydrodynamic sizes and fluorescence intensities. Figures 1 and 2 of ref 13 report apparent exponents that differ at 99% confidence, assuming normality. We revisit Figure 1 of ref 13, while Figure 2 of ref 13 reports an apparent exponent of 3.04 ± 0.09. A comparison of apparent and reference sizes indicated relative errors of the mean of 4% and of the standard deviation of almost 40%. Equation 3 corrects the exponent to 5.45 ± 0.16, changing the conclusion of the study from volumetric to supervolumetric scaling. Simulations indicate good accuracy for this correction if the sizing errors are truly multiplicative, but an undercorrection of nearly 10% on average if the sizing errors are truly additive (Figure S6).

These two studies^10,13^ measured fluorescent nanoparticle standards of similar size and manufacture, suggesting that different methods should yield a similar property–size correlation. Measurement error models achieve provisional consistency of the corrected exponents, harmonizing previously discordant conclusions.

Other studies show comparable sizing errors that could affect correlative analysis.^14^ For example, holography of the diffusion of polystyrene and silica nanoparticles provided image data to a neural network to infer refractive index and size. A comparison of apparent and reference sizes yielded consistent means and a relative error of the standard deviation of 75%, which a model fit of the size distribution reduced to 21% (Figures 3 and S3 of ref 14, Table 1). An apparent negative dependence of refractive index difference on nanoparticle size lacked a correlative model. For illustration, we can estimate the hypothetical impact of sizing errors on a power-law exponent with a negative value. Application of eq 3 yields correction factors of 3.1 and 1.47 for relative errors of the mean of 0% and −0.4%, and relative errors of the standard deviation of 75% and 21%, respectively. Simulations indicate good accuracy for both corrections (Figure S7).

Still in the first category are studies^13,15^ in which the sizing errors present an uncertain form that precludes the reliable application of our corrections.

Figure 1 of ref 13 reports an apparent exponent of 3.35 ± 0.08, which slightly exceeds 3 at high confidence, assuming normality. This supervolumetric exponent, even after attenuation, is self-inconsistent with the conclusion of ref 13. Simulations indicate good accuracy of the correction factor of 5.3 for a relative error of the mean of –10% and a relative error of the standard deviation of 107% with μ_ref_ = 25 nm and σ_ref_ = 2.75 nm, as in Figure 1 of ref 13 (Figure 2 and Table S4). A more complex error model is evidently necessary, however, as simulation results from either the PLE+ or PLE× model yield intensity values outside of the bounds of corresponding measurement results in Figure 1 of ref 13. A likely issue is that the measurement error could depend on the true particle size (Note S5, and Figures S10 and S11). The problem of correction inaccuracy due to an inaccurate model is concerning, as is mistaking an expected yet incorrect exponent for an unexpected yet correct exponent.

A chromatographic method^15^ shows even larger sizing errors and exponent corrections (Table S4), suggesting corrected exponents that would be so high as to give pause.

### Uncertainty Estimates

If reference sizes are unavailable for comparison to apparent sizes, then an estimate of sizing uncertainty^8,9^ can suffice to model sizing errors and correct exponent bias. However, two additional assumptions are necessary—that the mean size has zero bias, and that the uncertainty variance is exactly the excess variance of the apparent sizes. Adding detail to Figure 1, the process to determine PLE+ and PLE× corrections is as follows. Estimate the standard deviation that summarizes the radius uncertainty, σ_unc_, and the corresponding variance, σ_unc_^2^. For additive errors, assume σ_a_^2^ = σ_unc_^2^, calculate σ_ref_^2^ = σ_app_^2^ – σ_unc_^2^, where σ_app_ is the standard deviation of the apparent radii, assume μ_a_ = 0, and apply eq 1. For multiplicative errors, assume μ_m_ = 1, such that μ_app_ = μ_ref_, assume σ_m_^2^μ_ref_^2^ = σ_unc_^2^, calculate σ_ref_^2^ = σ_app_^2^ – σ_unc_^2^, and apply eq 2. For either PLE+ or PLE×, the corrected exponent is

4 $$\beta_{\mathrm{corr}}=\beta_{\mathrm{app}}\left[\frac{\sigma_{\mathrm{app}}^{2}}{\sigma_{\mathrm{app}}^{2}-\sigma_{\mathrm{unc}}^{2}}\right]$$

A limitation of this approach is that the accuracy of the correction factor depends on the accuracy of the uncertainty estimate. A comprehensive evaluation of measurement uncertainty^29,30,32^ would first identify and correct all errors that cause bias, and then propagate correction uncertainty along with all causes of measurement variability that contribute to the total uncertainty. Details that indicate such an evaluation are often absent from research reports and underestimates of uncertainty are common,^31^ which could lead to undercorrections of exponent attenuation. To address this issue, we study the effects of underestimating σ_unc_, as well as incorrectly assuming zero bias of the mean value (Note S6), deriving expressions that support examples and tests of future scenarios. Despite this limitation, we emphasize that the default alternative to an inaccurate correction is the questionable assumption of the outright equivalence of apparent and true values, as is the case for no correction.

One study tested Mie theory for gold nanoparticles,^8^ measuring steric size by scanning electron microscopy, and photothermal absorption and light scattering by optical microscopy. Considering the smallest particles, with an apparent mean radius of 25.5 nm, for which a power-law model of dependent optical properties is a good approximation (Figure 2 of ref 8), the reported sizing uncertainty of σ_unc_ = 2 nm would bias an expected exponent of 3 or 6 for absorption and scattering trends, respectively, requiring a hypothetical correction factor of 1.4 (Table 1). Simulations indicate good accuracy for this correction (Figure S8). Scanning electron microscopy is a mature method, and a discussion of several aspects of instrument calibration and uncertainty evaluation build confidence in this uncertainty estimate.^8^ Still, the projection of complex particle shapes into scanning electron micrographs and principal component analysis of such image data could bias the independent variable, while particle–substrate interactions could bias the dependent variable. Rather than fitting a power-law model to the data by least-squares estimation, this study scaled and plotted Mie theory predictions over a wide range of particle sizes.

Another study tested a hypothetical expectation of surface loading of fluorophores into lipid vesicles.^9^ This study measured the steric size of gold nanoparticles by scanning electron microscopy, as well as hydrodynamic size by nanoparticle tracking analysis with lipid bilayer tethering. The purpose was to test the latter method, which allowed correlation of the fluorescence intensity and hydrodynamic size of fluorescent vesicles. For the apparent distributions of vesicle size, the reported radius uncertainty of 4%, or σ_unc_ ≈ 1.43 nm, would only slightly attenuate a surface loading exponent (Table 1), and simulations indicate good correction accuracy (Figure S9). However, considering the potential for bias in methods that affect the surface structure of soft particles,^12^ the complexity of the tethering calibration, and the indications of additional measurement error in Supplementary Figure 4 of ref 9, this reported value could underestimate the total uncertainty. Again, rather than fitting a power-law model to the data by least-squares estimation, this study scaled and plotted a theoretical model with an exponent of two, evoking visual similarity to validate the results.

### No Corrections

The absence of either a reference size distribution or a sizing uncertainty estimate^20,21^ prohibits any correction of a power-law exponent. This lack of information limits the reliability of efforts to use an apparent power-law exponent either to validate sizing results or to infer particle size from intensity, such as of polymeric and semiconductor nanoparticles.^20,21^ In the latter scenario, the size inference could be biased, as follows.

We consider a hypothetical but relevant sizing scenario with no bias, excess variance, and a positive power-law exponent. Attenuation of the exponent would be apparent. After correction, the power-law model would still pass through the central tendency of the data. Therefore, without correction, for a low intensity below the mean, the size inference would be biased small. For an intensity close to the mean, per the hypothesis of the scenario, the size inference would be unbiased. For a high intensity above the mean, the size inference would be biased large. A schematic diagram of this scenario is in Figure S12. In other scenarios, such as a negative error of mean size, corresponding assessments are possible.

## Conclusions

We derive a generally applicable correction for least-squares estimates of correlative model parameters subject to measurement errors of the independent variable. Focusing on the prevalent power-law model for either additive or multiplicative errors, we present exponent corrections for representative studies of optical property–size correlations subject to sizing errors of nanoparticles. Our corrections impact the conclusions of several studies but only scratch the surface of what is likely to be a much deeper problem in nanoscience and nanotechnology, considering that parameter bias due to measurement error is so rife as to be an iron law^27^ or a persistent problem^33^ in other fields of study. For instance, beyond optical property–size correlations, which have broad implications, power-law exponents correlating the electrical and dimensional properties of nanowires^34,35^ may benefit from correction due to potential errors of wire size in experimental measurements. In this way, we present future opportunities to improve correlative accuracy, while also drawing attention to some outstanding challenges.

First and foremost, the sensitivity of parameter estimates to measurement errors of the independent variable is critical to understand and account for in nanoscale property–structure correlation. Seemingly small errors can cause surprisingly large biases.^22^ As a result, superficial consistency of apparent and expected exponents, such as an exponent of two for surface loading or three for volume loading with size as the independent variable,^9,12,13^ can give misleading insight. In the important case of polystyrene nanoparticles, which provide reference sizes and might also provide reference exponents for the size scaling of fluorescence intensity, any model of the intensity–size trend that considers only fluorophore loading and ignores the full scope of interactions of light with a dielectric nanoparticle containing a fluorophore ensemble is evidently insufficient.^10^ In this way, mature nanoparticle standards can still reveal unexpected optical phenomena, motivating further studies to address this issue.^11^

The second challenge is that measurement error models can improve accuracy but are still sensitive to assumptions, approximations, and uncertainties. Certified reference data are the most reliable source of information to determine measurement errors. However, even reference data for mature standards can have significant uncertainty that is dark or invisible within individual methods.^19,31^ Beyond bootstrap resampling of sampling variability, Monte Carlo methods can account for uncertainty of reference data that comes to light in intermethod comparisons. A critical issue is that many nanostructures lack standards and reference data altogether. In this common situation, an uncertainty estimate can suffice to determine a measurement error model, but an evaluation of uncertainty is itself challenging and often leads to an underestimate, which leads to an undercorrection. If a question remains as to whether it is better to correct and err, or not to correct but still err, then further study is necessary to obtain a reliable quantity.

More work is necessary to complete our partial solution to a fundamental problem. The ongoing development of nanostructure standards with reference data will enable the best models of measurement errors. Until then, best practices of uncertainty evaluation will allow good progress in approximating measurement errors to improve the accuracy of parameter values for correlative models. Otherwise, biases will continue to obscure the true correlation of nanoscale properties and sizes, for nanoparticles and other types of nanostructures.

## Methods

### Derivations

We derive custom method-of-moments corrections for the least-squares estimates of α and β from the general correlative model *f*(*y*) = α + β*g*(*x*) + δ. Our derivation begins by finding an approximate expression for the expected value of β_app_, the least-squares estimate of β. This expression is a function of β, the true value of the slope parameter in our general model, so solving the expression for β produces the requisite correction factor. The corrected estimate of α follows directly from the corrected estimate of β (Note S1).

### Simulations

We restrict our simulations to power-law models, *g*(·) = ln(·) and *f*(·) = ln(·), and choose values for α, β, μ_ref_, σ_ref_, ϵ_μ_μ_ref_^–1^, and ϵ_σ_σ_ref_^–1^ (Figures 2, 3, and S1–S9). First, the simulations draw reference values of the independent variable, *x*_ref_, from a normal distribution with a mean, μ_ref_, and a standard deviation, σ_ref_. We compare the results of drawing reference values from normal and log-normal distributions (Table S3). For each value of *x*_ref_, the simulations draw a value of *f*(*y*) from a normal distribution with a mean of α + β*g*(*x*_ref_) and a standard deviation of 0.05. Then, the simulations determine the values of μ_a_ and σ_a_ or μ_m_ and σ_m_ from μ_ref_, σ_ref_, ϵ_μ_μ_ref_^–1^, and ϵ_σ_σ_ref_^–1^ as necessary. Each value of *x*_ref_ involves the calculation of *x*_app_ as *x*_app_ = *x*_ref_ + ϵ_a_ or *x*_app_ = ϵ_m_ *x*_ref_, drawing ϵ_a_ or ϵ_m_ from a normal distribution with mean μ_a_ or μ_m_ and standard deviation σ_a_ or σ_m_. Next, the simulations perform least-squares regression on g­(*x*_app_) and *f*(*y*) to determine β_app_ and then β_corr_ from eqs 1–3. Last, the simulations return the quantity of interest, such as the relative error of the correction, ϵ_β_corr_β_app_^–1^_ = (β_corr_ – β)β^–1^ (Figure 2d–i). All figures and tables presenting simulation results involve the average of many simulations.

### Digitization

We digitize plots using the methods of ref 22, approximating the digitization uncertainty as the process repeatability for a standard operator of the digitizer software.

### Uncertainty

We evaluate uncertainty by Monte Carlo methods, performing parametric bootstrap resampling of statistical sampling variability as possible. We report all uncertainties as 68% coverage intervals (Note S4).

## Supplementary Material





## Notes

Code and Data (ZIP)
Terminology; correction derivation; measurement function and uncertainty evaluation; exemplary corrections using synthetic data; synthetic data for exemplary corrections; additive errors of mean values; multiplicative errors of mean values; multiplicative errors of standard deviation values; correction summary for μ_ref_ = 50 nm and σ_ref_ = 5 nm; correction summary for Table 1 of reference 10; correction summary for Figure 2 of ref 13; correction summary for Figures 3 and S3 of ref 14; correction summary for Figure 1 of ref 8; correction summary for Figure 5 of ref 9; distributional effects on exponent corrections; evaluation of refs 8–10 and 13–15 ; coverage intervals and regions; model assumptions; corrections for Figure 1 of ref 13; corrections for Figure 2 of ref 13; correction errors due to incorrect assumptions; size inference from apparent exponent (PDF)
