# Supplementary material for: Measurement Error Models Enable Accurate Correlation of Nanoscale Properties and Structures
Source: ACS Nano. 2026 Aug 6;20(32):22381–90. doi: 10.1021/acsnano.5c12141 (PMC13492464; doi:10.1021/acsnano.5c12141)
Supplement: Supplementary file 1 [file nn5c12141_si_002.pdf]

Supporting Information

Measurement Error Models Enable Accurate Correlation of Nanoscale Properties and Structures

Adam L. Pintar,[†, ⊥] Andrew C. Madison,[‡, ⊥] Craig R. Copeland,[‡] Natalia Farkas,[‡, §, ‖] and Samuel M. Stavis [‡, *]

Index

Table S1	Terminology
Note S1	Correction Derivation
Note S2	Measurement Function and Uncertainty Evaluation
Note S3	Exemplary Corrections Using Synthetic Data
Table S2	Synthetic Data for Exemplary Corrections
Figure S1	Additive Errors of Mean Values
Figure S2	Multiplicative Errors of Mean Values
Figure S3	Multiplicative Errors of Standard Deviation Values
Figure S4	Correction Summary for $\mu_{ref} = 50$ nm and $\sigma_{ref} = 5$ nm
Figure S5	Correction Summary for Table 1 of Reference 10
Figure S6	Correction Summary for Figure 2 of Reference 13
Figure S7	Correction Summary for Figures 3 and S3 of Reference 14
Figure S8	Correction Summary for Figure 1 of Reference 8
Figure S9	Correction Summary for Figure 5 of Reference 9
Table S3	Distributional Effects on Exponent Corrections
Table S4	Evaluation of References 8–10 and 13–15
Note S4	Coverage Intervals and Regions
Note S5	Model Assumptions
Figure S10	Corrections for Figure 1 of Reference 13
Figure S11	Corrections for Figure 2 of Reference 13
Note S6	Correction Errors due to Incorrect Assumptions
Figure S12	Size Inference from Apparent Exponent
References	

[†] Statistical Engineering Division, National Institute of Standards and Technology, Gaithersburg, Maryland 20899, United States
[‡] Microsystems and Nanotechnology Division, National Institute of Standards and Technology, Gaithersburg, Maryland 20899, United States
[§] Theiss Research, La Jolla, California 92037, United States
[‖] Building and Fire Sciences, United States Forest Service, Forest Products Laboratory, Madison, Wisconsin 53726, United States
[⊥] Equal contributions
* sstavis@nist.gov

Table S1. Terminology

Term	Definition	Footnote
$f(y) = \alpha + \beta g(x) + \delta$	General correlative model	
y	Dependent variable of a correlative model, such as optical intensity	
$f(y)$	Function that linearizes the relationship of independent and dependent variables	
α	True vertical-axis intercept, such as the natural log of the true coefficient of a power-law model	
β	True slope, such as the true exponent of a power-law model	
$g(x)$	Function that linearizes the relationship of independent and dependent variables	
x	True independent variable of a correlative model, such as particle size	
δ	Additive error of the dependent variable	
x_{app}	Apparent value of the independent variable of a correlative model, such as particle size	
i	Index for a unique member of a statistical sample from a population under test	
ξ	Input to a measurement function	
M	Index for inputs to a measurement function	
$m(\xi_1, \ldots, \xi_M)$	Measurement function with M inputs	
x_{ref}	Reference measurement of the independent variable of a correlative model, such as particle size	
μ	General term for a mean value	
σ	General term for a standard deviation	
N	Statistical sample size for an apparent measurement result	
N_{ref}	Statistical sample size for a reference measurement result	
$\mathrm{E}[\cdot]$	Expectation value of the random variable inside the square brackets	
μ_{app}	$\mathrm{E}[x_{\text{app}}] \approx \frac{1}{N}\sum_i x_{\text{app}_i}$	*a*
μ_{ref}	$\mathrm{E}[x] = \mathrm{E}[x_{\text{ref}}] \approx \frac{1}{N_{\text{ref}}}\sum_i x_{\text{ref}_i}$	*a*
$\mathrm{Var}[\cdot]$	Variance of the random variable inside the square brackets	
σ_{app}	$\sqrt{\mathrm{Var}[x_{\text{app}}]} \approx \sqrt{\frac{1}{N-1}\sum_i (x_{\text{app}_i} - \mu_{\text{app}})^2}$	*b*
σ_{ref}	$\sqrt{\mathrm{Var}[x_{\text{ref}}]} \approx \sqrt{\frac{1}{N_{\text{ref}}-1}\sum_i (x_{\text{ref}_i} - \mu_{\text{ref}})^2}$	*b*
$\overline{f(y)}$	$\frac{1}{N}\sum_i f(y_i)$	
$\overline{g(x_{\text{app}})}$	$\frac{1}{N}\sum_i g(x_{\text{app}_i})$	
$S_{f g_{\text{app}}}$	$\frac{1}{N}\sum_i [f(y_i) - \overline{f(y)}][g(x_{\text{app}_i}) - \overline{g(x_{\text{app}})}]$	*c*
$S_{g_{\text{app}} g_{\text{app}}}$	$\frac{1}{N}\sum_i \left[g(x_{\text{app}_i}) - \overline{g(x_{\text{app}})}\right]^2$	*d*

Continues on next page

Table S1. Terminology—continued

Term	Definition	Footnote
β_{app}	$S_{f g_{\text{app}}} S^{-1}_{g_{\text{app}} g_{\text{app}}}$	e
β_{corr}	Corrected least-squares estimate of β	
σ_{unc}	Estimate of standard uncertainty	
$h(x, \epsilon)$	Measurement error model	
ϵ	General symbol representing error for the independent variable	
ϵ_{a}	Additive error for the independent variable	
ϵ_{m}	Multiplicative error for the independent variable	
δ_{trans}	e^{δ}	
α_{trans}	e^{α}	
μ_{a}	$\text{E}[\epsilon_{\text{a}}]$	
σ_{a}	$\sqrt{\text{Var}[\epsilon_{\text{a}}]}$	
μ_{m}	$\text{E}[\epsilon_{\text{m}}]$	
σ_{m}	$\sqrt{\text{Var}[\epsilon_{\text{m}}]}$	
ϵ_{μ}	$\mu_{\text{app}} - \mu_{\text{ref}}$	
ϵ_{σ}	$\sigma_{\text{app}} - \sigma_{\text{ref}}$	
$\epsilon_{\beta_{\text{corr}}\beta^{-1}_{\text{app}}}$	$(\beta_{\text{corr}} - \beta)/\beta$	
p	Polynomial degree	
$\text{Cov}[\cdot,\cdot]$	Covariance of the random variables inside the square brackets	
$g'(x)$	The derivative of g at x	
α_{corr}	Corrected scaling coefficient of power-law model	

[a] The left side of this approximation is the theoretical or population mean value. The right side of this approximation is the statistical sample mean value. We intend the former for a theoretical derivation and the latter for an experimental result.
[b] The left side of this approximation is the theoretical or population standard deviation. The right side of this approximation is the statistical sample standard deviation. We intend the former for a theoretical derivation and the latter for an experimental result.
[c] This expression is proportional to the statistical sample covariance between the transformed dependent and independent variables.
[d] This expression is proportional to the statistical sample variance of the transformed independent variable.
[e] Apparent least-squares estimate of β.

Note S1. Correction Derivation

Introduction. Equations 1 and 2 resemble the correction that is implicit in eq 1.1.6 in ref 25, because the measurement error models from which these equations arise are similar. In ref 25, using the notation of Table S1, regression of y on x involves only additive errors of x with a mean value of zero. In the present study, for the PLE+

model, regression of $\ln(y)$ on $\ln(x)$ involves additive errors with a mean value that is potentially non-zero for x. Our derivation yields the same correction that is implicit in eq 1.1.6 of ref 25, but with an extra factor that is a function of the mean values of the error and reference distributions. The multiplicative model works more naturally with regression of $\ln(y)$ on $\ln(x)$, yielding essentially the same formula that is implicit in eq 1.1.6 of ref 25, but replacing variances of the reference and error distributions with variances of logarithmic transformations of random variables with distributions that match the reference and error distributions.

Before performing a logarithmic transformation of measurement results with explicit units, it is necessary to divide the measurement results by a reference quantity to create dimensionless ratios. For a power-law model, the choice of the reference quantity affects interpretation of the coefficient but not the exponent. In the present study, we set all reference quantities to one unit of the measurement results.

Reference 23 presents further developments of measurement error models and explicitly considers method-of-moments corrections for regressing $\ln(y)$ on $\ln(x)$. However, there are some differences between the corrections in ref 23 and our derivations below. Most importantly, our correction considers a general relationship between y and x, as well as a general measurement error model. Reference 23 considers several non-linear relationships between the dependent and independent variables, but only additive measurement errors of mean zero, which cannot describe the cancellation effect between the mean and variance of the error distribution that we previously observed.[22]

Derivation. We consider relationships of the form $f(y) = \alpha + \beta g(x) + \delta$, where y is the dependent variable, α is the vertical-axis intercept, β is the regression slope, x is the independent variable, and δ is a random variable representing an additive error for the dependent variable after transformation by $f(y)$, which linearizes the relationship along with the function $g(x)$. This general form applies to several important models, as follows.

First, a power-law model with a multiplicative error, $y = \delta_{\text{trans}}\alpha_{\text{trans}}x^{\beta}$, implies equivalent logarithmic transformation functions $f(\cdot) = \ln(\cdot)$ and $g(\cdot) = \ln(\cdot)$, such that $\alpha = \ln(\alpha_{\text{trans}})$ and $\delta = \ln(\delta_{\text{trans}})$. Similarly, for $y = (\alpha + \beta x + \delta)^{p}$, which is a polynomial relationship with an error variance that depends on the value of the independent variable, $y^{\frac{1}{p}} = \alpha + \beta x + \delta$, such that $f(\cdot) = (\cdot)^{\frac{1}{p}}$ and $g(\cdot) = (\cdot)$. Models of this form are useful if a power transformation of only the dependent variable linearizes the relationship between y and x and stabilizes the error variance. Other common models that accommodate this form are the exponential model with a multiplicative error, $y = \delta_{\text{trans}}\alpha_{\text{trans}}e^{\beta x}$, and the simple linear model with additive error, $y = \alpha + \beta x + \delta$. In addition to these

transformations, we consider general measurement error models of the form $x_{\text{app}} = h(x, \epsilon)$, where x_{app} are apparent values of x subject to measurement error. Common forms include the additive error model, $x_{\text{app}} = h(x, \epsilon_{\text{a}}) = x + \epsilon_{\text{a}}$, and the multiplicative error model, $x_{\text{app}} = h(x, \epsilon_{\text{m}}) = \epsilon_{\text{m}} x$. In the following derivation, for clarity, we use x, the true value of the independent variable instead of x_{ref}, the reference measurement of it, making the distinction only at the end of the derivation.

Deriving the approximate correction for least-squares estimates of β begins by taking the expectation of the multivariate Taylor series expansion of the apparent slope parameter β_{app} around the point ($\text{E}[S_{f g_{\text{app}}}]$, $\text{E}[S_{g_{\text{app}} g_{\text{app}}}]$), where $S_{f g_{\text{app}}}$ is proportional to the sample covariance of the N pairs of $f(y)$ and $g(x_{\text{app}})$, and $S_{g_{\text{app}} g_{\text{app}}}$ is proportional to the sample variance of the N values of $g(x_{\text{app}})$. The expectation yields

$$\text{E}[\beta_{\text{app}}] \approx \frac{\text{E}\left[S_{f g_{\text{app}}}\right]}{\text{E}\left[S_{g_{\text{app}} g_{\text{app}}}\right]} \tag{S1}$$

Starting with the denominator of eq S1, $\text{E}\left[S_{g_{\text{app}} g_{\text{app}}}\right] \approx \text{Var}\left[g\left(x_{\text{app}}\right)\right] \approx \left[g'\left(\text{E}[x_{\text{app}}]\right)\right]^2 \text{Var}[x_{\text{app}}]$, where g' is the derivative of g with respect to its single argument. For the numerator, $E\left[S_{f g_{\text{app}}}\right] \approx \beta \text{Cov}[g(x), g\left(x_{\text{app}}\right)]$. Using the Taylor-series expansion of $g(x_{\text{app}})$ around x, $g\left(x_{\text{app}}\right) \approx g(x) + g'(x)(x_{\text{app}} - x)$, we obtain $\text{Cov}\left[g(x), g\left(x_{\text{app}}\right)\right] \approx \text{Var}[g(x)] + \text{Cov}\left[g(x), g'(x)\left(x_{\text{app}} - x\right)\right] \approx [g'(\text{E}[x])]^2 \text{Var}[x] + \text{Cov}[g(x), g'(x)\left(x_{\text{app}} - x\right)]$. Substituting these approximations into eq S1 yields an approximation for the expected apparent correlation parameter

$$\text{E}\left[\beta_{\text{app}}\right] \approx \frac{\beta\{[g'(\text{E}[x])]^2 \text{Var}[x] + \text{Cov}\left[g(x), g'(x)\left(x_{\text{app}} - x\right)\right]\}}{\left[g'\left(\text{E}[x_{\text{app}}]\right)\right]^2 \text{Var}[x_{\text{app}}]} \tag{S2}$$

Solving eq S2 for β yields a general correction

$$\beta \approx \frac{\beta_{\text{app}}\left[g'\left(\text{E}[x_{\text{app}}]\right)\right]^2 \text{Var}[x_{\text{app}}]}{[g'(\text{E}[x])]^2 \text{Var}[x] + \text{Cov}\left[g(x), g'(x)(x_{\text{app}} - x)\right]} = \beta_{\text{corr}} \tag{S3}$$

For completeness, a corrected estimate of the intercept, α, is $\alpha_{\text{corr}} = \overline{f(y)} - \beta_{\text{corr}} g(\text{E}[x])$.

Power-Law Exponent Subject to Additive Errors (PLE+). For the PLE+ model, $g(\cdot) = \ln(\cdot)$ and $x_{\text{app}} = h(x, \epsilon_{\text{a}}) = x + \epsilon_{\text{a}}$, with $\text{E}[\epsilon_{\text{a}}] = \mu_{\text{a}}$ and $\text{Var}[\epsilon_{\text{a}}] = \sigma_{\text{a}}^2$. Assuming $\text{E}[x] = \text{E}[x_{\text{ref}}] = \mu_{\text{ref}}$ and $\text{Var}[x] = \text{Var}[x_{\text{ref}}] = \sigma_{\text{ref}}^2$ implies

$$g'\left(\text{E}\left[x_{\text{app}}\right]\right) = \frac{1}{\text{E}[x] + \text{E}[\epsilon_{\text{a}}]} = \frac{1}{\mu_{\text{ref}} + \mu_{\text{a}}} \tag{S4}$$

$$g'(\mathrm{E}[x]) = \frac{1}{\mathrm{E}[x]} = \frac{1}{\mu_{\mathrm{ref}}} \tag{S5}$$

$$\mathrm{Var}\left[x_{\mathrm{app}}\right] = \mathrm{Var}[x] + \mathrm{Var}[\epsilon_{\mathrm{a}}] = \sigma_{\mathrm{ref}}^2 + \sigma_{\mathrm{a}}^2 \tag{S6}$$

$$\mathrm{Cov}\left[g(x), g'(x)(x_{\mathrm{app}} - x)\right] \approx \frac{-\mu_{\mathrm{a}}\sigma_{\mathrm{ref}}^2}{\mu_{\mathrm{ref}}^3} \tag{S7}$$

Substituting eqs S4, S5, S6, and S7 into eq S3 yields

$$\beta_{\mathrm{corr}} = \beta_{\mathrm{app}} \frac{\dfrac{\sigma_{\mathrm{ref}}^2 + \sigma_{\mathrm{a}}^2}{(\mu_{\mathrm{ref}} + \mu_{\mathrm{a}})^2}}{\dfrac{\sigma_{\mathrm{ref}}^2}{\mu_{\mathrm{ref}}^2} - \dfrac{\mu_{\mathrm{a}}\sigma_{\mathrm{ref}}^2}{\mu_{\mathrm{ref}}^3}} \approx \beta_{\mathrm{app}} \left[\frac{\sigma_{\mathrm{ref}}^2 + \sigma_{\mathrm{a}}^2}{\sigma_{\mathrm{ref}}^2}\right]\left[1 + \frac{\mu_{\mathrm{a}}}{\mu_{\mathrm{ref}}}\right]^{-2} = \beta_{\mathrm{app}} \frac{\sigma_{\mathrm{app}}^2\mu_{\mathrm{ref}}^2}{\sigma_{\mathrm{ref}}^2\mu_{\mathrm{app}}^2} \tag{S8}$$

after neglecting $\mu_{\mathrm{a}}\sigma_{\mathrm{ref}}^2/\mu_{\mathrm{ref}}^3$, which we expect to be small in comparison to $\sigma_{\mathrm{ref}}^2/\mu_{\mathrm{ref}}^2$, and noting that $\mu_{\mathrm{app}} = \mathrm{E}\left[x_{\mathrm{app}}\right] = \mu_{\mathrm{ref}} + \mu_{\mathrm{a}}$ and that $\sigma_{\mathrm{app}}^2 = \mathrm{Var}\left[x_{\mathrm{app}}\right] = \sigma_{\mathrm{ref}}^2 + \sigma_{\mathrm{a}}^2$ for $h(x, \epsilon_{\mathrm{a}}) = x + \epsilon_{\mathrm{a}}$. Retaining $\mu_{\mathrm{a}}\sigma_{\mathrm{ref}}^2/\mu_{\mathrm{ref}}^3$ could improve the accuracy of the correction for non-zero values of μ_{a} (Figures 2d–f) at the cost of a more complex expression for the PLE+ correction that deviates from the PLE≈ correction.

Power-Law Exponent Subject to Multiplicative Errors (PLE×). For the PLE× model, $g(\cdot) = \ln(\cdot)$ and $x_{\mathrm{app}} = h(x, \epsilon_{\mathrm{m}}) = \epsilon_{\mathrm{m}}x$, with $\mathrm{E}[\epsilon_{\mathrm{m}}] = \mu_{\mathrm{m}}$ and $\mathrm{Var}[\epsilon_{\mathrm{m}}] = \sigma_{\mathrm{m}}^2$. Assuming $\mathrm{E}[x] = \mathrm{E}[x_{\mathrm{ref}}] = \mu_{\mathrm{ref}}$ and $\mathrm{Var}[x] = \mathrm{Var}[x_{\mathrm{ref}}] = \sigma_{\mathrm{ref}}^2$ implies

$$g'\left(\mathrm{E}\left[x_{\mathrm{app}}\right]\right) = \frac{1}{\mathrm{E}[\epsilon_{\mathrm{m}}]\mathrm{E}[x]} = \frac{1}{\mu_{\mathrm{m}}\mu_{\mathrm{ref}}} \tag{S9}$$

$$g'(\mathrm{E}[x]) = \frac{1}{\mathrm{E}[x]} = \frac{1}{\mu_{\mathrm{ref}}} \tag{S10}$$

$$\mathrm{Var}\left[x_{\mathrm{app}}\right] \approx \mathrm{E}^2[\epsilon_{\mathrm{m}}]\mathrm{Var}[x] + \mathrm{E}^2[x]\mathrm{Var}[\epsilon_{\mathrm{m}}] = \mu_{\mathrm{m}}^2\sigma_{\mathrm{ref}}^2 + \mu_{\mathrm{ref}}^2\sigma_{\mathrm{m}}^2 \tag{S11}$$

$$\mathrm{Cov}\left[g(x), g'(x)(x_{\mathrm{app}} - x)\right] = 0 \tag{S12}$$

Substituting eqs S9, S10, S11, and S12 into eq S3 yields

$$\beta_{\mathrm{corr}} = \beta_{\mathrm{app}} \frac{\dfrac{\mu_{\mathrm{m}}^2\sigma_{\mathrm{ref}}^2 + \mu_{\mathrm{ref}}^2\sigma_{\mathrm{m}}^2}{(\mu_{\mathrm{m}}\mu_{\mathrm{ref}})^2}}{\dfrac{\sigma_{\mathrm{ref}}^2}{\mu_{\mathrm{ref}}^2} - 0} = \beta_{\mathrm{app}} \frac{\dfrac{\sigma_{\mathrm{ref}}^2}{\mu_{\mathrm{ref}}^2} + \dfrac{\sigma_{\mathrm{m}}^2}{\mu_{\mathrm{m}}^2}}{\dfrac{\sigma_{\mathrm{ref}}^2}{\mu_{\mathrm{ref}}^2}} \approx \beta_{\mathrm{app}} \frac{\sigma_{\mathrm{app}}^2\mu_{\mathrm{ref}}^2}{\sigma_{\mathrm{ref}}^2\mu_{\mathrm{app}}^2} \tag{S13}$$

where the last approximation leverages the equality $\mu_{\text{app}} = \mathrm{E}[x_{\text{app}}] = \mu_{\text{m}}\mu_{\text{ref}}$ and the additional approximation $\sigma_{\text{app}}^2 = \mathrm{Var}[x_{\text{app}}] \approx \mu_{\text{m}}^2\sigma_{\text{ref}}^2 + \mu_{\text{ref}}^2\sigma_{\text{m}}^2$.

Replacing eq S11 with $\mathrm{Var}[x_{\text{app}}] = \mu_{\text{m}}^2\sigma_{\text{ref}}^2 + \mu_{\text{ref}}^2\sigma_{\text{m}}^2 + \sigma_{\text{m}}^2\sigma_{\text{ref}}^2$ could improve accuracy for studies involving relatively broad distributions of the independent variable. For relatively narrow distributions of the independent variable, the replacement expression adds a third term, $\sigma_{\text{m}}^2\sigma_{\text{ref}}^2/\mu_{\text{m}}^2\mu_{\text{ref}}^2$, to the numerator of eq S13. Because this third term is the product of the first two terms, each of which is typically less than unity, it should be comparatively small. This replacement would change the correction in eq 2, increasing its complexity, but eq 3 remains valid.

Note S2. Measurement Function and Uncertainty Evaluation

Evaluation of Uncertainty. If reference data are unavailable, an evaluation of measurement uncertainty can suffice to model measurement errors. The best practice is to express a measurement function and propagate input uncertainties through the function to an output uncertainty. Several examples of this process are in refs 29 and 32. The simplest, though often implicit, function is an identity expression. In this minimal function, an input random variable propagates directly to an output random variable, with a normal distribution modeling measurement repeatability. A less obvious but still important issue is the need to correct bias and propagate any correction uncertainty in the process of reducing source data to calibrated quantities. To model correction uncertainty, possible elaborations on the identity function include incorporating additive or multiplicative random variables with normal distributions.

Forms of Expression. An analytic expression of a measurement function, such as a rearrangement of the Stokes–Einstein equation for particle sizing, allows for an evaluation of sizing uncertainty first by the law of propagation of uncertainty to build insight, and then by Monte Carlo simulations to avoid the approximations of the former method. However, complex measurement processes, such as nanoparticle tracking analysis with microfluidic confinement correction, may involve numerical computation that is difficult to express in an analytic form. To our knowledge, a complete evaluation of uncertainty does not yet exist for this measurement process. For this reason, in Figure 1, we depict a symbolic measurement function to propagate input uncertainties through a generic variant of the Stokes–Einstein equation to an output uncertainty. The Ishikawa diagram is a similar form of expression.

Note S3. Exemplary Corrections Using Synthetic Data

Synthetic Data. Synthetic data for exemplary corrections are in Table S2, with the following columns. The first column is the statistical sample number or metric. The second column is a statistical sample of reference radii, x_{ref}, that a simulation draws from a normal distribution with a mean of 25 nm and a variance of (2.5 nm)^2. The third column is the natural logarithm of the second column. The fourth column is a statistical sample of apparent radii, x_{app}, from a normal distribution with a mean of 25 nm and a variance of (2.5 nm)^2 + (2.5 nm)^2, showing an excess variance of (2.5 nm)^2 but no bias of mean radius. There are more reference radii than apparent radii, emphasizing that these statistical samples correspond to different particles. The fifth column is the natural logarithm of the fourth column. The sixth column is the natural logarithm of fluorescence intensity, y, for the same particles with apparent radii. The values of y are from a normal distribution with a mean of –14.3 ln(arb. / 1 arb.) + 6 ln(x), where x is the true radius, and a variance of [0.25 ln(arb. / 1 arb.)]^2. The values of x are absent from Table S2 because x is unknowable in real measurements. For completeness, the true radii are draws from a normal distribution with a mean of 25 nm and a variance of (2.5 nm)^2. The true exponent is $\beta = 6$ for the following examples.

Available Reference Data. The apparent exponent resulting from least-squares estimation is a severe attenuation of the true exponent, $\beta_{\text{app}} = \text{Cov}\left[y, \ln(x_{\text{app}})\right]/\text{Var}\left[\ln(x_{\text{app}})\right] \approx 0.0671/0.0182 = 3.69 < \beta = 6$. The numerator of this expression is calculable from the data in the fifth and six columns of Table S2, and the denominator is calculable from the data in the fifth column. Using the data in the second and third columns of Table S2, the correction factor from eqs S8 and S13 is $\left(\sigma_{\text{app}}^2 \mu_{\text{ref}}^2\right)/\left(\sigma_{\text{ref}}^2 \mu_{\text{app}}^2\right) \approx ([3.24\text{ nm}]^2 \times [26.06\text{ nm}]^2)/([2.74\text{ nm}]^2 \times [24.46\text{ nm}]^2) = 1.59$. The corrected exponent is $\beta_{\text{corr}} \approx 1.59 \times 3.69 = 5.87$.

Unavailable Reference Data. Even without reference data, such that the second and third columns of Table S2 are unavailable, a correction of the apparent exponent, $\beta_{\text{app}} \approx 3.69$, is possible on the basis of an evaluation of measurement uncertainty. If such an evaluation were to yield the exact value of excess variance, $\sigma_{\text{unc}}^2 = (2.5\text{ nm})^2$, then $\beta_{\text{corr}} = \beta_{\text{app}}\, \sigma_{\text{app}}^2/\left(\sigma_{\text{app}}^2 - \sigma_{\text{unc}}^2\right) \approx 3.69 \times (3.24\text{ nm})^2/([3.24\text{ nm}]^2 - [2.5\text{ nm}]^2) = 9.12$.

Sampling Variability. The striking discrepancy between the corrected exponents is due to statistical sampling variability, which causes a small undercorrection in the example with available reference data and a large overcorrection in the example with unavailable reference data. Thus, small statistical samples facilitate calculation, emphasize the detrimental effect of sampling variability, and motivate larger statistical samples in experiments.

Table S2. Synthetic Data for Exemplary Corrections

Statistical sample number or metric	Reference particle radius x_{ref} (nm)	Natural logarithm of reference particle radius $\ln(x_{ref})$ (ln [nm / 1 nm])	Apparent particle radius x_{app} (nm)	Natural logarithm of apparent particle radius $\ln(x_{app})$ (ln [nm / 1 nm])	Natural logarithm of optical intensity y (ln [arb. / 1 arb.])
1	24.41	3.19	26.17	3.26	5.08
2	26.37	3.27	24.31	3.19	4.32
3	25.25	3.23	22.14	3.1	4.88
4	29.19	3.37	26.6	3.28	5.45
5	26.24	3.27	24.14	3.18	6.14
6	27.87	3.33	24.05	3.18	3.82
7	29.37	3.38	26.49	3.28	5.74
8	25.31	3.23	25.4	3.23	5.21
9	24.15	3.18	22.33	3.11	4.65
10	25.38	3.23	18.84	2.94	3.89
11	27.11	3.3	21.26	3.06	4.75
12	27.26	3.31	18.57	2.92	4.32
13	24.3	3.19	26.32	3.27	4.9
14	22.53	3.11	29.9	3.4	6.32
15	27.57	3.32	23.58	3.16	4.42
16	22.38	3.11	20.47	3.02	4.44
17	32.02	3.47	25.57	3.24	5.03
18	30.82	3.43	25.96	3.26	5.21
19	28.04	3.33	31.14	3.44	5.56
20	25.82	3.25	25.95	3.26	6.15
21	22.34	3.11	–	–	–
22	21.87	3.09	–	–	–
23	25.13	3.22	–	–	–
24	28.54	3.35	–	–	–
25	22.29	3.1	–	–	–
Mean	26.06	3.26	24.46	3.19	5.01
St. dev.	2.74	0.10	3.24	0.1351 [a]	0.72

[a] Four decimal places preserve accuracy in the calculation of β_{app}.

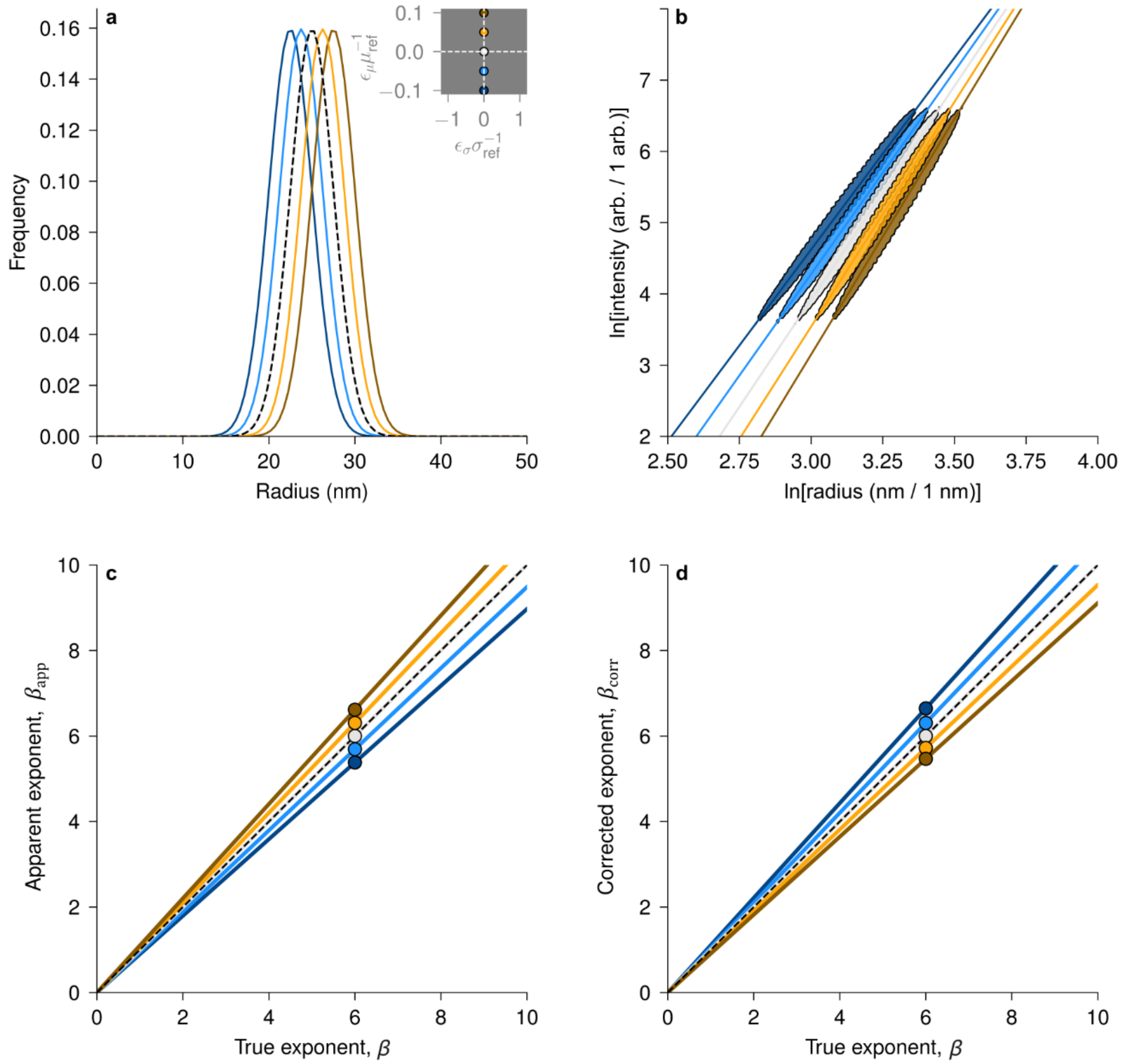

Figure S1. Additive Errors of Mean Values. (a) Probability densities showing (black dash) the reference radius distribution with $\mu_{ref} = 25$ nm and $\sigma_{ref} = 2.5$ nm subject to (shades of blue, white, and orange) additive errors of $\epsilon_\mu \mu_{ref}^{-1}$ ranging from –10% to 10% and $\epsilon_\sigma \sigma_{ref}^{-1} = 0\%$. (a, inset) Map showing relative errors of the standard deviation, $\epsilon_\sigma \sigma_{ref}^{-1}$, and relative errors of the mean, $\epsilon_\mu \mu_{ref}^{-1}$, with respect to the reference values. (b) Plots showing joint histograms of ln[intensity (arb. / 1 arb.)] versus ln[radius (nm / 1 nm)] for a true exponent of $\beta = 6$, subject to the additive errors of radius in (a) and additive errors of ln[intensity (arb. / 1 arb.)] of zero mean and a standard deviation of 0.05 ln(arb. / 1 arb.). Solid lines result from least-squares regression. Elliptical regions cover 95% of these errors. (c, d) Plots showing (c) apparent exponents and (d) corrected exponents as a function of true exponent for the errors of the mean in (a). Circles in (c) and (d) correspond to a true exponent of $\beta = 6$. Line thicknesses in (c) and (d) cover 68% of statistical sampling variability. Black dash lines in (c) and (d) indicate an equality of true and apparent or corrected exponents.

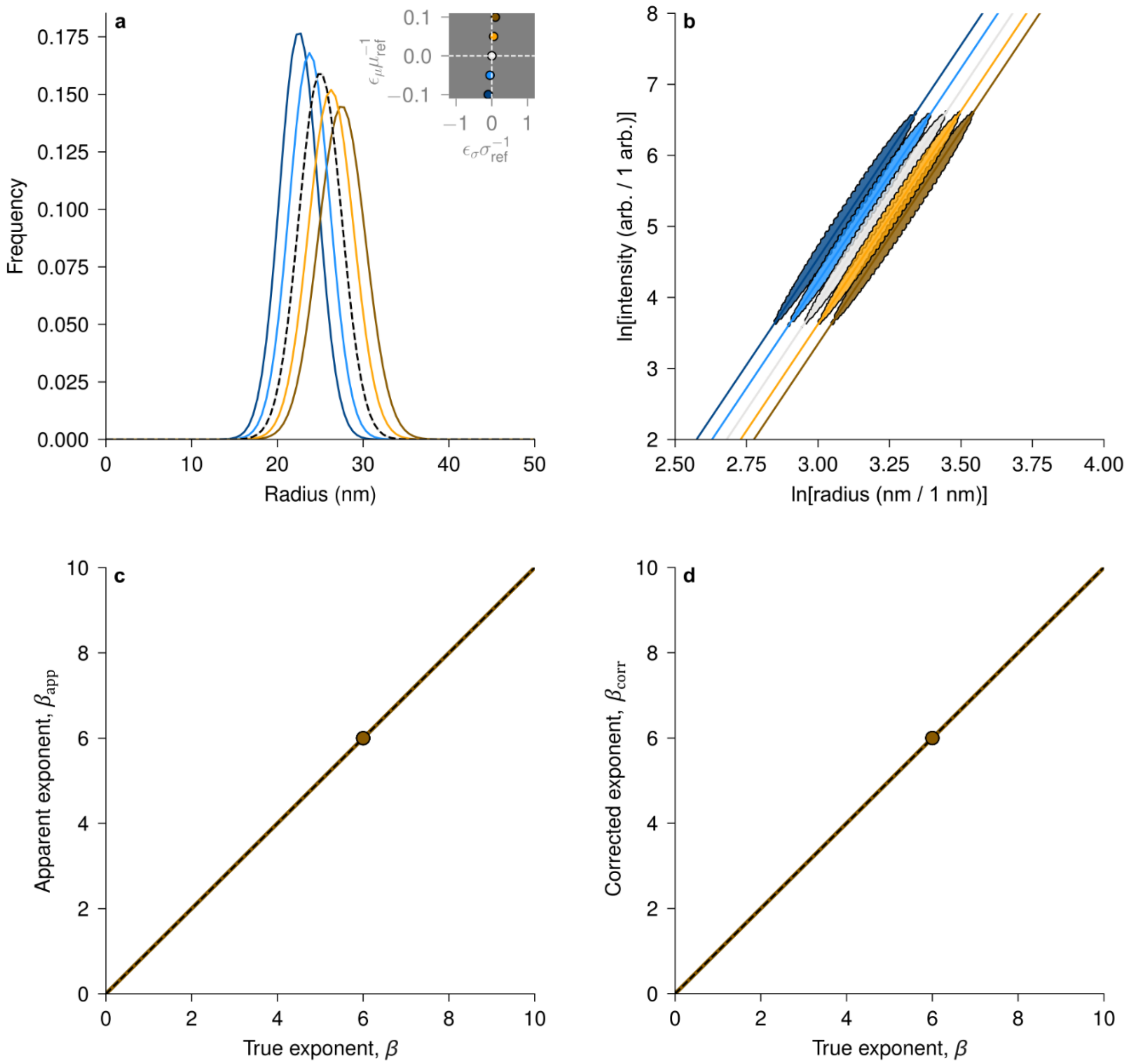

Figure S2. Multiplicative Errors of Mean Values. (a) Probability densities showing (black dash) the reference radius distribution with $\mu_{\mathrm{ref}} = 25$ nm and $\sigma_{\mathrm{ref}} = 2.5$ nm subject to (shades of blue, white, and orange) multiplicative errors with $\epsilon_\mu \mu_{\mathrm{ref}}^{-1}$ ranging from -10% to 10% and $\epsilon_\sigma \sigma_{\mathrm{ref}}^{-1} = \epsilon_\mu \mu_{\mathrm{ref}}^{-1}$. (a, inset) Map showing relative errors of the standard deviation, $\epsilon_\sigma \sigma_{\mathrm{ref}}^{-1}$, and relative errors of the mean, $\epsilon_\mu \mu_{\mathrm{ref}}^{-1}$, with respect to the reference values. (b) Plots showing joint histograms of ln[intensity (arb. / 1 arb.)] versus ln[(radius (nm / 1 nm)] for a true exponent of $\beta = 6$, subject to the multiplicative errors of radius in (a) and additive errors of ln[intensity (arb. / 1 arb.)] of zero mean and a standard deviation of 0.05 ln(arb. / 1 arb.). Solid lines result from least-squares regression. Elliptical regions cover 95% of these errors. (c, d) Plots showing (c) apparent exponents and (d) corrected exponents as a function of true exponent for the errors in (a). Circles in (c) and (d) correspond to a true exponent of $\beta = 6$. Line thicknesses in (c) and (d) cover 68% of statistical sampling variability. Black dash lines in (c) and (d) indicate an equality of true and apparent or corrected exponents.

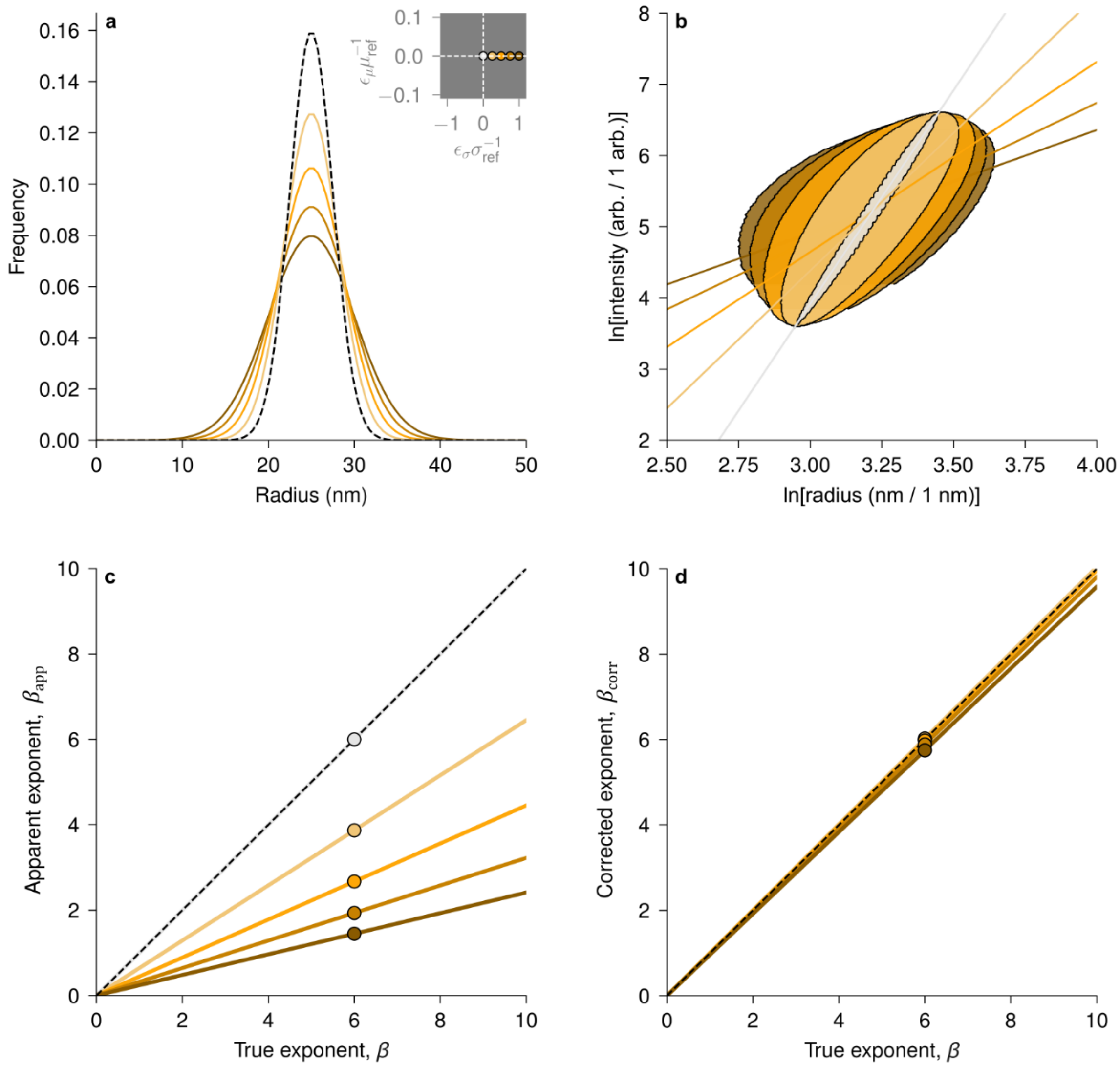

Figure S3. Multiplicative Errors of Standard Deviation Values. (a) Probability densities showing (black dash) the reference radius distribution with $\mu_{\mathrm{ref}} = 25$ nm and $\sigma_{\mathrm{ref}} = 2.5$ nm and subject to (shades of orange) multiplicative errors with $\epsilon_\mu \mu_{\mathrm{ref}}^{-1} = 0\%$ and $\epsilon_\sigma \sigma_{\mathrm{ref}}^{-1}$ ranging from 0% to 100%. (a, inset) Map showing relative errors of the standard deviation, $\epsilon_\sigma \sigma_{\mathrm{ref}}^{-1}$, and relative errors of the mean, $\epsilon_\mu \mu_{\mathrm{ref}}^{-1}$, with respect to the reference values. (b) Plots showing joint histograms of ln[intensity (arb. / 1 arb.)] versus ln[radius (nm / 1 nm)] for a true exponent of $\beta = 6$, subject to the multiplicative errors of radius in (a) and additive errors of ln[intensity (arb. / 1 arb.)] of zero mean and a standard deviation of 0.05 ln(arb. / 1 arb.). Solid lines result from least-squares regression. Elliptical and oblong regions cover 95% of these errors. (c, d) Plots showing (c) apparent exponents and (d) corrected exponents as a function of true exponent for the errors in (a). Circles in (c) and (d) correspond to a true exponent of $\beta = 6$. Line thicknesses in (c) and (d) cover 68% of statistical sampling variability. Black dash lines in (c) and (d) indicate an equality of true and apparent or corrected exponents.

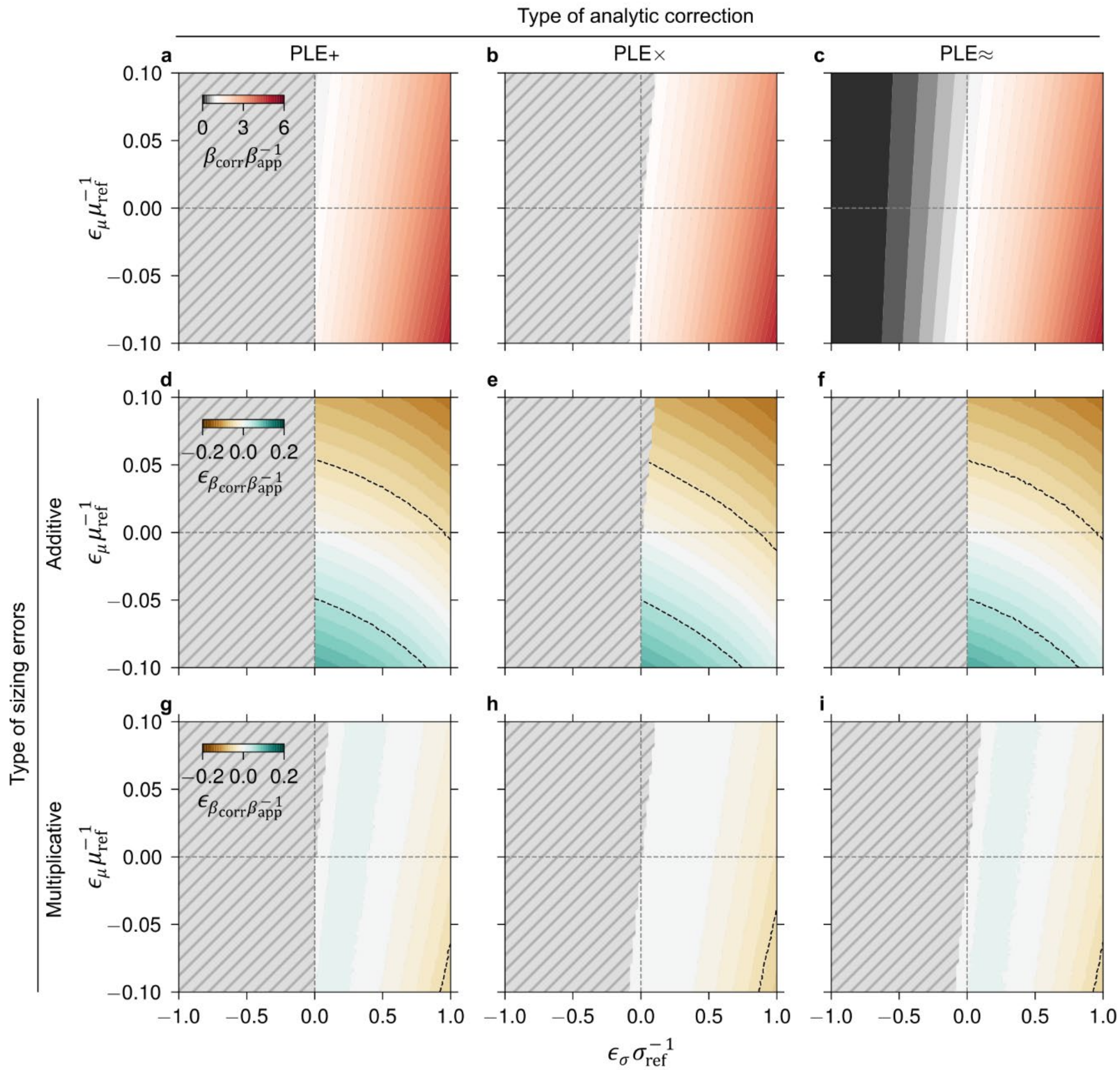

Figure S4. Correction Summary for $\mu_{\mathrm{ref}} = 50$ nm and $\sigma_{\mathrm{ref}} = 5$ nm. (a–c) Contour plots showing three types of correction factors for least-squares estimates of power-law exponents. (d–i) Contour plots showing a matrix of relative errors of the correction factors for (d–f) additive and (g–i) multiplicative sizing errors. The plots show (a, d, g) PLE+, (b, e, h) PLE×, and (c, f, i) PLE≈ corrections as functions of the relative error of the standard deviation, $\epsilon_\sigma \sigma_{\mathrm{ref}}^{-1}$, and the relative error of the mean, $\epsilon_\mu \mu_{\mathrm{ref}}^{-1}$ with $\mu_{\mathrm{ref}} = 50$ nm and $\sigma_{\mathrm{ref}} = 5$ nm. Gray hatch regions are invalid parameter combinations. Black dash contours in (d–i) bound a relative error of the correction factors of ±5%. The data marker is the measurement result from Table 1 of ref 10.

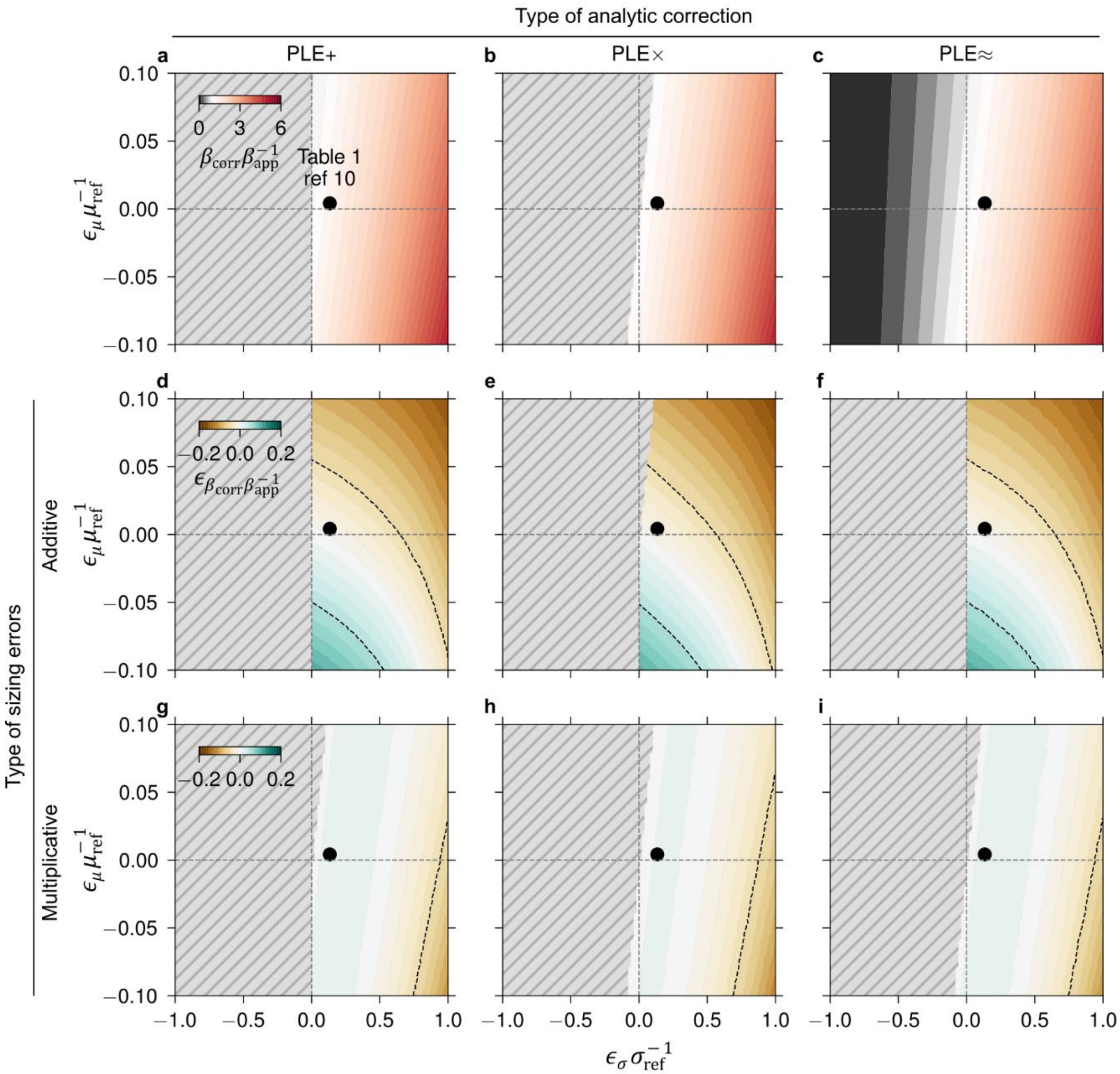

Figure S5. Correction Summary for Table 1 of Reference 10. (a–c) Contour plots showing three types of correction factors for least-squares estimates of power-law exponents. (d–i) Contour plots showing a matrix of relative errors of the correction factors for (d–f) additive and (g–i) multiplicative sizing errors. The plots show (a, d, g) PLE+, (b, e, h) PLE×, and (c, f, i) PLE≈ corrections as functions of the relative error of the standard deviation, $\epsilon_\sigma \sigma_{ref}^{-1}$, and the relative error of the mean, $\epsilon_\mu \mu_{ref}^{-1}$ with $\mu_{ref} = 24$ nm and $\sigma_{ref} = 3$ nm. Gray hatch regions are invalid parameter combinations. Black dash contours in (d–i) bound a relative error of the correction factors of ±5%. The data marker is the measurement result from Table 1 of ref 10.

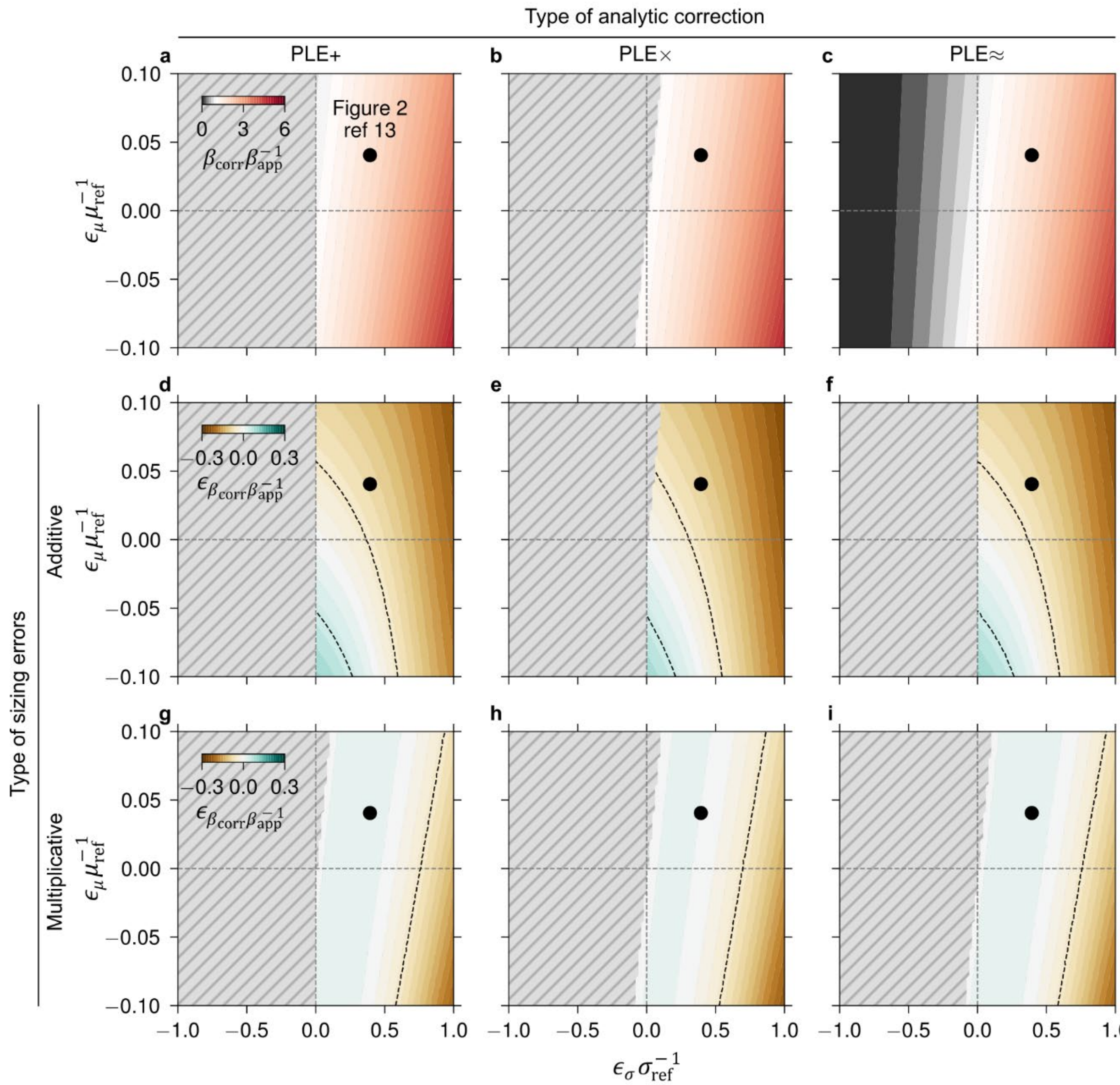

Figure S6. Correction Summary for Figure 2 of Reference 13. (a–c) Contour plots showing three types of correction factors for least-squares estimates of power-law exponents. (d–i) Contour plots showing a matrix of relative errors of the correction factors for (d–f) additive and (g–i) multiplicative sizing errors. The plots show (a, d, g) PLE+, (b, e, h) PLE×, and (c, f, i) PLE≈ corrections as functions of the relative error of the standard deviation, $\epsilon_\sigma \sigma_{\mathrm{ref}}^{-1}$, and the relative error of the mean, $\epsilon_\mu \mu_{\mathrm{ref}}^{-1}$ with $\mu_{\mathrm{ref}} = 18.5$ nm and $\sigma_{\mathrm{ref}} = 3.1$ nm. Gray hatch regions are invalid parameter combinations. Black dash contours in (d–i) bound a relative error of the correction factors of ±5%. The data marker is the measurement result from Figure 2 of ref 13.

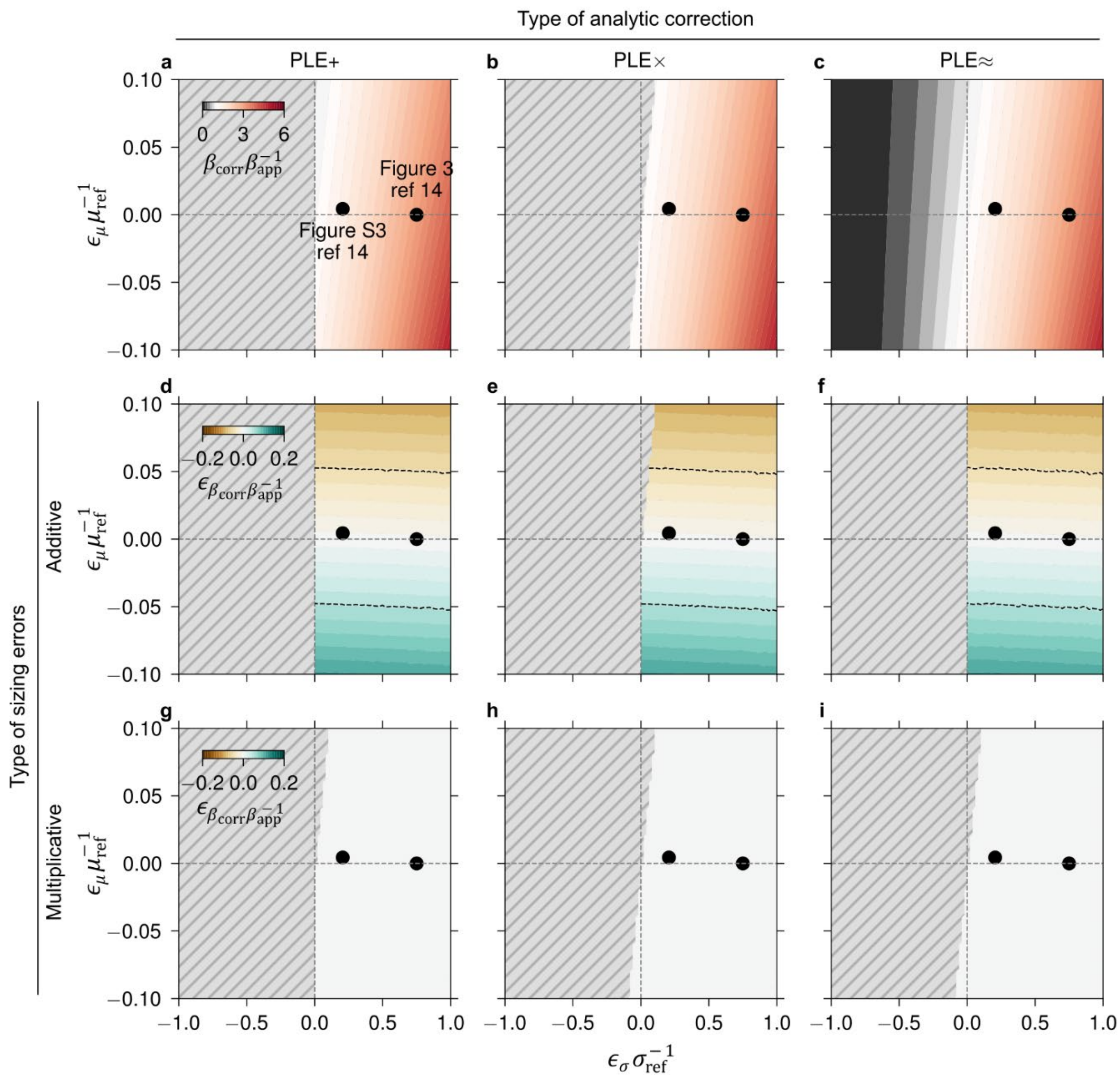

Figure S7. Correction Summary for Figures 3 and S3 of Reference 14. (a–c) Contour plots showing three types of correction factors for least-squares estimates of power-law exponents. (d–i) Contour plots showing a matrix of relative errors of the correction factors for (d–f) additive and (g–i) multiplicative sizing errors. The plots show (a, d, g) PLE+, (b, e, h) PLE×, and (c, f, i) PLE≈ corrections as functions of the relative error of the standard deviation, $\epsilon_\sigma \sigma_{ref}^{-1}$, and the relative error of the mean, $\epsilon_\mu \mu_{ref}^{-1}$ with $\mu_{ref} = 228$ nm and $\sigma_{ref} = 6.8$ nm. Gray hatch regions are invalid parameter combinations. Black dash contours in (d–i) bound a relative error of the correction factors of ±5%. The data markers are the measurement results from Figures 3 and S3 of ref 14.

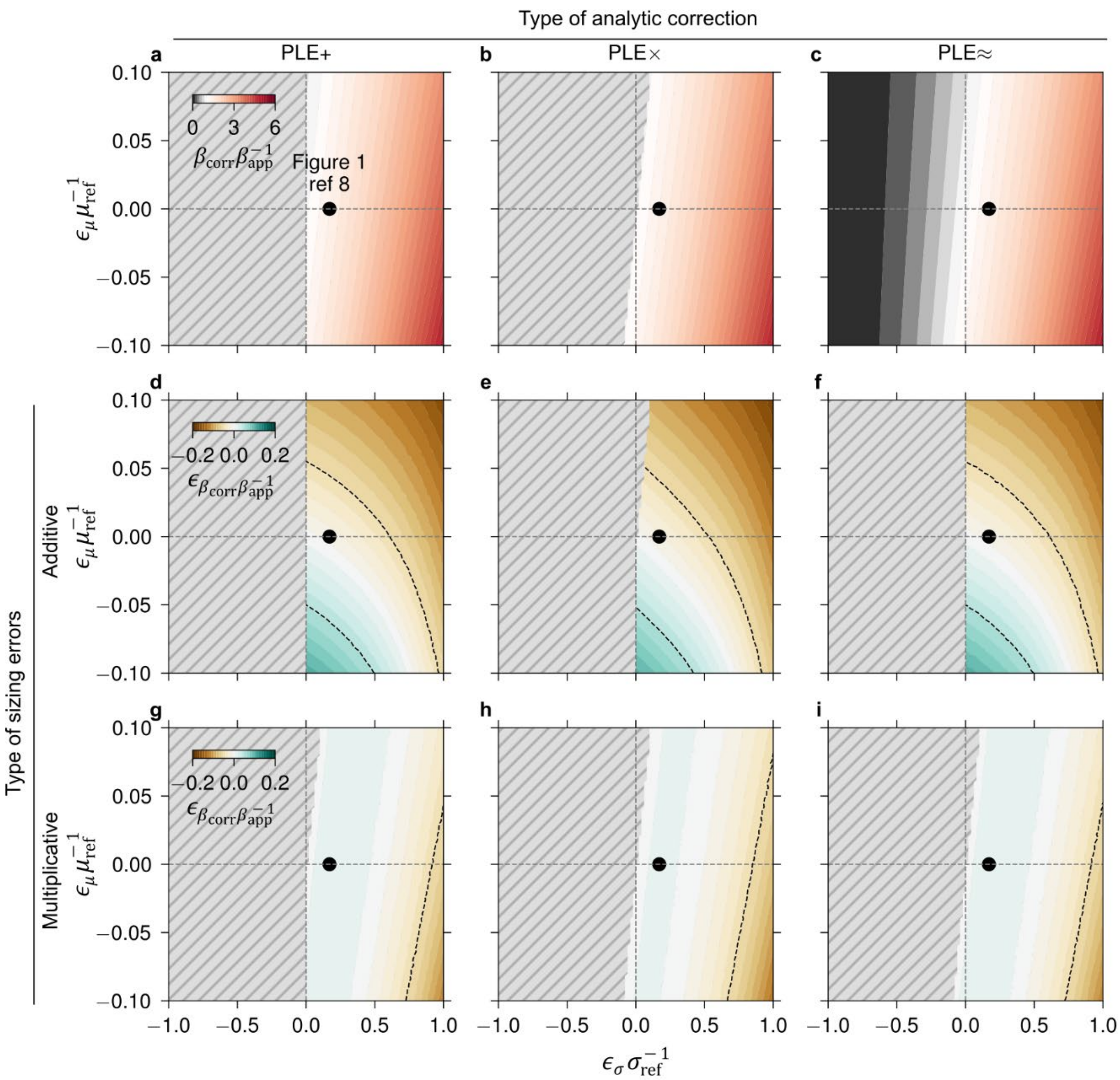

Figure S8. Correction Summary for Figure 1 of Reference 8. (a–c) Contour plots showing three types of correction factors for least-squares estimates of power-law exponents. (d–i) Contour plots showing a matrix of relative errors of the correction factors for (d–f) additive and (g–i) multiplicative sizing errors. The plots show (a, d, g) PLE+, (b, e, h) PLE×, and (c, f, i) PLE≈ corrections as functions of the relative error of the standard deviation, $\epsilon_\sigma \sigma_{\mathrm{ref}}^{-1}$, and the relative error of the mean, $\epsilon_\mu \mu_{\mathrm{ref}}^{-1}$ with $\mu_{\mathrm{ref}} = 25.5$ nm and $\sigma_{\mathrm{ref}} = 3.3$ nm. Gray hatch regions are invalid parameter combinations. Black dash contours in (d–i) bound a relative error of the correction factors of ±5%. The data markers are the measurement results from Figures 1 of ref 8.

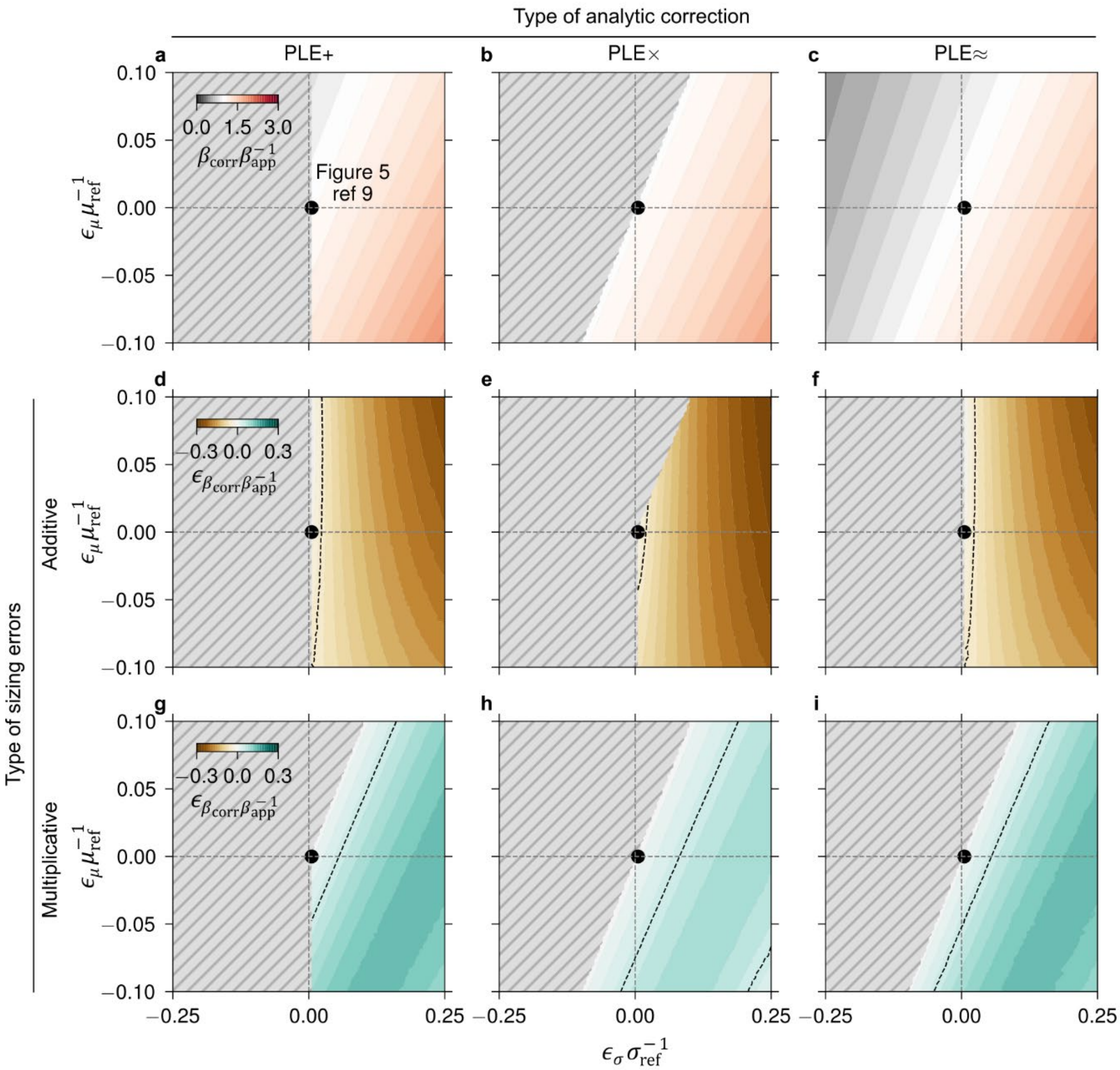

Figure S9. Correction Summary for Figure 5 of Reference 9. (a–c) Contour plots showing three types of correction factors for least-squares estimates of power-law exponents. (d–i) Contour plots showing a matrix of relative errors of the correction factors for (d–f) additive and (g–i) multiplicative sizing errors. The plots show (a, d, g) PLE+, (b, e, h) PLE×, and (c, f, i) PLE≈ corrections as functions of the relative error of the standard deviation, $\epsilon_\sigma \sigma_{\text{ref}}^{-1}$, and the relative error of the mean, $\epsilon_\mu \mu_{\text{ref}}^{-1}$ with $\mu_{\text{ref}} = 35.6$ nm and $\sigma_{\text{ref}} = 14$ nm. Gray hatch regions are invalid parameter combinations. Black dash contours in (d–i) bound a relative error of the correction factors of ±5%. The data markers are the measurement results from Figure 5 of ref 9.

Table S3. Distributional Effects on Exponent Corrections[a, b]

Source	Reference distribution	Error model	β (–)	β_{app} (–)	Difference (%)
Figure 1 of ref 13	Normal	PLE+	18.0	3.36	2.7
Figure 1 of ref 13	Lognormal	PLE+	18.0	3.27	
Figure 1 of ref 13	Normal	PLE×	18.0	3.08	2.9
Figure 1 of ref 13	Lognormal	PLE×	18.0	2.99	
Figure 1 of ref 13	Normal	Gumbel	18.0	3.25	2.2
Figure 1 of ref 13	Lognormal	Gumbel	18.0	3.18	
Figure 2 of ref 13	Normal	PLE+	5.5	2.81	3.6
Figure 2 of ref 13	Lognormal	PLE+	5.5	2.71	
Figure 2 of ref 13	Normal	PLE×	5.5	3.13	3.8
Figure 2 of ref 13	Lognormal	PLE×	5.5	3.01	
Figure 2 of ref 13	Normal	Gumbel	5.5	3.03	3.6
Figure 2 of ref 13	Lognormal	Gumbel	5.5	2.92	

[a] Simulations involving Gumbel errors include true exponents from manual selection rather than analytic correction (Note S5).
[b] All simulations involve a statistical sample that is overwhelmingly large, such that uncertainty in β_{app} is negligibly small.

Table S4. Evaluation of References 8–10 and 13–15[a]

Source	μ_{ref} (nm)	σ_{ref} (nm)	σ_{unc} (nm)	μ_{app} (nm)	σ_{app} (nm)	$\epsilon_\mu \mu_{\text{ref}}^{-1}$ (–)	$\epsilon_\sigma \sigma_{\text{ref}}^{-1}$ (–)	$\beta_{\text{corr}} \beta_{\text{app}}^{-1}$ (–)	β_{app} (–)	β_{corr} (–)
Figure 1 of ref 8[b, c, d]	–	–	2	25.5	3.8	–	0.17	1.4	–	–
Figure 4 of ref 9[b, c, e]	–	–	1.43 ± 0.01	35.6 ± 0.2	14.1 ± 0.4	–	0.0052 ± 0.0002	1.0104 ± 0.0005	–	–
Table 1 of ref 10[f]	24	3	–	24.1 ± 0.1	3.40 ± 0.07	0.004 ± 0.004	0.13 ± 0.02	1.27 ± 0.05	4.0 ± 0.1	5.10 ± 0.25
Figure 1 of ref 13[c]	25	2.75	–	22.5	5.7	-0.1	1.07	5.3	3.4 ± 0.1	18.0 ± 0.5
Figure 2 of ref 13[c]	18.5	3.05	–	19.25	4.25	0.04	0.39	1.79	3.04 ± 0.09	5.45 ± 0.16
Figure 3 of ref 14[b, c, g]	228	6.8	–	–	11.9 ± 0.5	–	0.75 ± 0.08	3.1 ± 0.3	–	–
Figure S3 of ref 14[b, c, g]	228	6.8	–	227	8.2	-0.004	0.21	1.47	–	–
Figure S10 of ref 15[b, h]	20	2.25	–	49.4 ± 0.3	13.1 ± 0.3	1.47 ± 0.02	4.84 ± 0.15	5.6 ± 0.3	–	–

[a] Mean and standard deviation values correspond to radii. All uncertainties are 68% coverage intervals (Note S4). Corrections are from eq 3 or 4 as appropriate. In these notes, we summarize several issues that arise in obtaining, interpreting, and comparing quantities from non-uniform reports. The main issues are: the availability of different amounts of information; the use of non-specific terms such as peak; and the reporting of uncertainty estimates of various types and coverage factors.

[b] References 8, 9, and 14 present property–size correlations without least-squares fits of power-law models, so corresponding values of β_{app} and β_{corr} are unavailable. Reference 15 omits a property–size correlation in the test of a size standard, which results in sizing errors that are most suitable for the present calculation of an exponent correction. Similar corrections could apply to Figure 4d of ref 15.

[c] References 8, 9, and 13 omit statistical sample sizes, so the corresponding sampling uncertainties of μ_{app} and σ_{app} are unavailable. Figure 3 of ref 14 reports a statistical sample size of 256 particles, whereas Figure S3 of ref 14 analyzes only the central feature of the radius distribution and omits the statistical sample size.

[d] Reference 8 reports average experimental size distributions of 15% and a sizing uncertainty of 4 nm. Information to specify the size distribution in the inset of Figure 1a of ref 8 is unavailable.

[e] Figures 4 and 5 of ref 9 report peak radii of around 38 nm but omit the standard deviations of the radius distributions. To obtain σ_{app}, which requires a consistent μ_{app}, we digitize Figure 4b of ref 9, using the methods of ref 22. The corresponding uncertainties of μ_{app} and σ_{app} cover 68% of digitization repeatability. Reference 9 also reports a relative random error of 4% for radius.

[f] We reduce the statistical sampling uncertainties and coverage factors of ref 10 for direct comparison.

[g] Figure 3 of ref 14 omits the mean of the radius distribution, whereas Figure S3 of ref 14 reports the peak position of a normal fit of the radius distribution, which we interpret as its mean.

[h] Figure S10c of ref 15 reports a number average size of 97.0 nm and a dynamic light scattering (DLS) polydispersity index of 0.119. To obtain consistent values of μ_{app} and σ_{app} from the derived diameter histogram, we digitize Figure S10c of ref 15, using the methods of ref 22. The corresponding uncertainties cover 68% of statistical sampling uncertainty and digitization repeatability.

Note S4. Coverage Intervals and Regions

We report all uncertainties as 68% coverage intervals to simplify comparison of results from various publications, accounting for different causes of uncertainty, depending on the availability of corresponding information. In contrast, to show how measurement errors affect our simulation results in Figures 3b, S1b, S2b, and S3b, we depict elliptical and oblong regions that cover the central 95% of joint histograms of $\ln[\text{intensity (arb. / 1 arb.)}]$ versus apparent $\ln[\text{radius (nm / 1 nm)}]$.

Note S5. Model Assumptions

The simulations of the present study build on computational methods that we developed previously.[22] For simplicity, our previous methods assumed the PLE+ model and normal distributions of reference sizes and measurement results. These assumptions result in normal distributions of measurement errors, which may deviate from experimental results for several reasons. A simplistic distributional model is one possible cause of deviation. Sizing errors for which the error distribution depends on the true size are also possible. We extend our previous methods to test both issues, including lognormal distributions of reference data and different types of measurement error models. With regard to the latter issue, we test three distinct models for their ability to approximate some particular measurement results from nanoparticle tracking analysis.[13]

First, the distributional approximations could deviate from both reference sizes and sizing results, which would carry through to measurement simulations and could affect the accuracy of the corrections. However, simulations modeling the reference radii by either normal or lognormal distributions show only a small effect of the distribution type for a representative study,[13] reducing the corresponding least-squares estimates of power-law exponents by no more than a few percent (Table S3). This test of our modeling assumptions would be most rigorous if the actual values of both the reference sizes and sizing results were available. In this case, computational deconvolution and resampling methods[22] could address the issue of distributional approximations.

Second, additive and multiplicative error models are often realistic but may be simplistic for some measurement processes. The PLE+ and PLE× models imply decreasing correlation of intensity and size as sizing errors broaden the true size distribution, forming the apparent size distribution. To an extent, this assumption is valid as excess variability could bias the intensity–size correlation and parameter estimates thereof. However, the effects of sizing errors could be more complex. Neither the PLE+ nor the PLE× model accounts for heteroscedasticity that appears to be present in some particular results from nanoparticle tracking analysis.[13] In a correlation of fluorescence intensity and particle size, heteroscedasticity could manifest in joint histograms of $\ln[\text{intensity (arb. / 1 arb.)}]$ versus $\ln[\text{radius (nm / 1 nm)}]$ that are wider for smaller, dimmer nanoparticles and narrower for larger, brighter nanoparticles. Physical concepts can partially explain this phenomenon. To begin, nanoparticles of increasing size tend to emit an increasing number of signal photons. While the present study addresses this very issue, the exact dependence of fluorescence intensity on nanoparticle size remains uncertain. As fluorescence intensity is the source of signal photons, this trend causes lower

uncertainty of both intensity and localization analyses, with the latter uncertainty affecting the apparent trajectory. It is more certain that nanoparticles of increasing size move more slowly than smaller nanoparticles, per the inverse proportionality of size and diffusivity. In this way, Brownian motion results in relatively less motion blur and resulting position uncertainty for larger nanoparticles, although the manifestation of diffusivity—which is lower for larger particles—is what allows for tracking analysis. Further study is necessary to fully understand how total sizing uncertainty varies with particle size, but at least some causes of sizing uncertainty decrease as particle size increases.

In a first step toward empirically modeling such effects, we identify and apply a Gumbel copula to model sizing errors with an adjustable parameter that controls the correlation between apparent and reference sizes and allows more variability for small reference sizes. This supporting model tests the corresponding effects on least-squares estimation of some particular measurement results.[13] For the result of ref 13 showing larger sizing errors, the Gumbel copula shows better agreement between the simulation and measurement results than either the PLE+ or PLE× models (Figure S10). Further, the Gumbel copula uses a true exponent of 10 in the simulations in comparison to 18 for the PLE+ and PLE× models (Table S3). For the result of ref 13 showing smaller sizing errors, the Gumbel copula simulates scatter that is consistent with the measurement results, as well as the PLE+ and PLE× models, using a true exponent of 5.5 in all simulations (Figure S11 and Table S3). These simulation results suggest a corrected exponent of approximately 5.5, which is consistent for all of the measurement error models that we consider.

Despite these complications, the simulation results in this study are fit for their primary purpose, which is to test our corrections for least-squares estimates of power-law exponents, rather than to exactly reproduce the sizing errors of a particular method or explain the significant differences between the sizing errors and corrected exponents of ref 13. We conclude that further study is necessary to fully understand the potential effects of distributional approximations and to develop more complex models of measurement errors for particular methods.

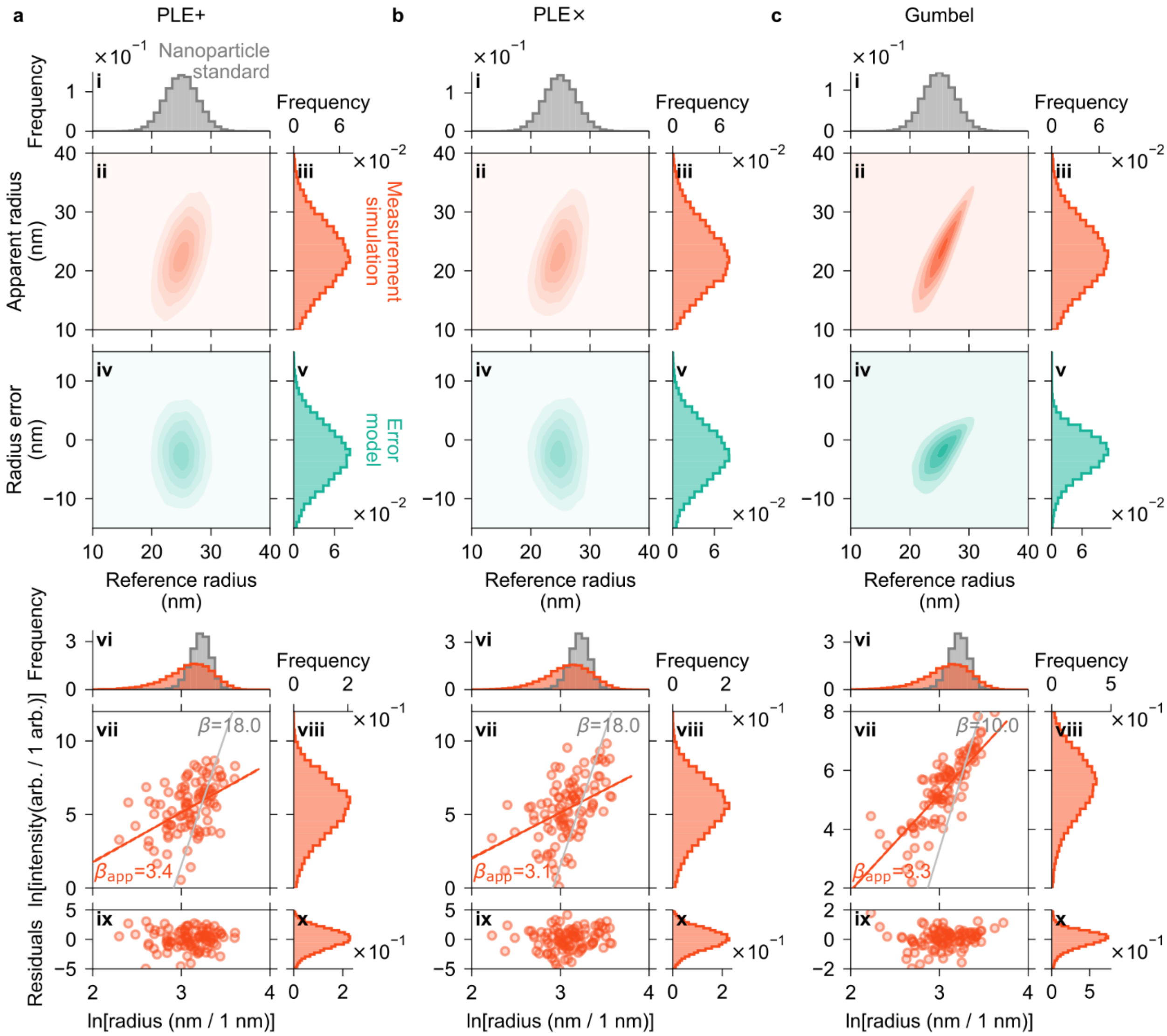

Figure S10. Corrections for Figure 1 of Reference 13. (a) PLE+, (b) PLE×, and (c) Gumbel models of errors of diameter apparent in Figure 1 of ref 13. (i–iii) Marginal and joint histograms showing correlations of reference and apparent radii. The joint histogram in (c, ii) results from sampling the bivariate Gumbel copula with a correlation parameter of $\theta = 3$. (iv–v) Joint and marginal histograms showing correlations of reference radius and radius errors. (vi–x) Marginal histograms and scatter plots showing biases that are evident as attenuation of an exponent or equivalent slope of a linear model of ln[intensity (arb. / 1 arb.)] versus ln[radius (nm / 1 nm)]. (vi) Marginal histograms showing (solid gray) ln[radius (nm / 1 nm)] data without measurement errors and (orange) apparent ln[radius (nm / 1 nm)] data approximating those of Figure 1 of ref 13. (vii) Scatter plot showing (orange circles) ln[intensity (arb. / 1 arb.)] versus ln[radius (nm / 1 nm)] data and (dark orange) least squares line and (gray) true line used for simulation. True exponents in (a, vii) and (b, vii) are approximately from eq 3, whereas the correction in (c, vii) is from manual selection to approximately match the scatter in Figure 1 of ref 13. (viii) Marginal histogram showing ln[intensity (arb. / 1 arb.)] measurements. (ix) Scatter plot and (x) marginal histogram showing residual values of ln[intensity (arb. / 1 arb.)] from the least squares fit in (vii).

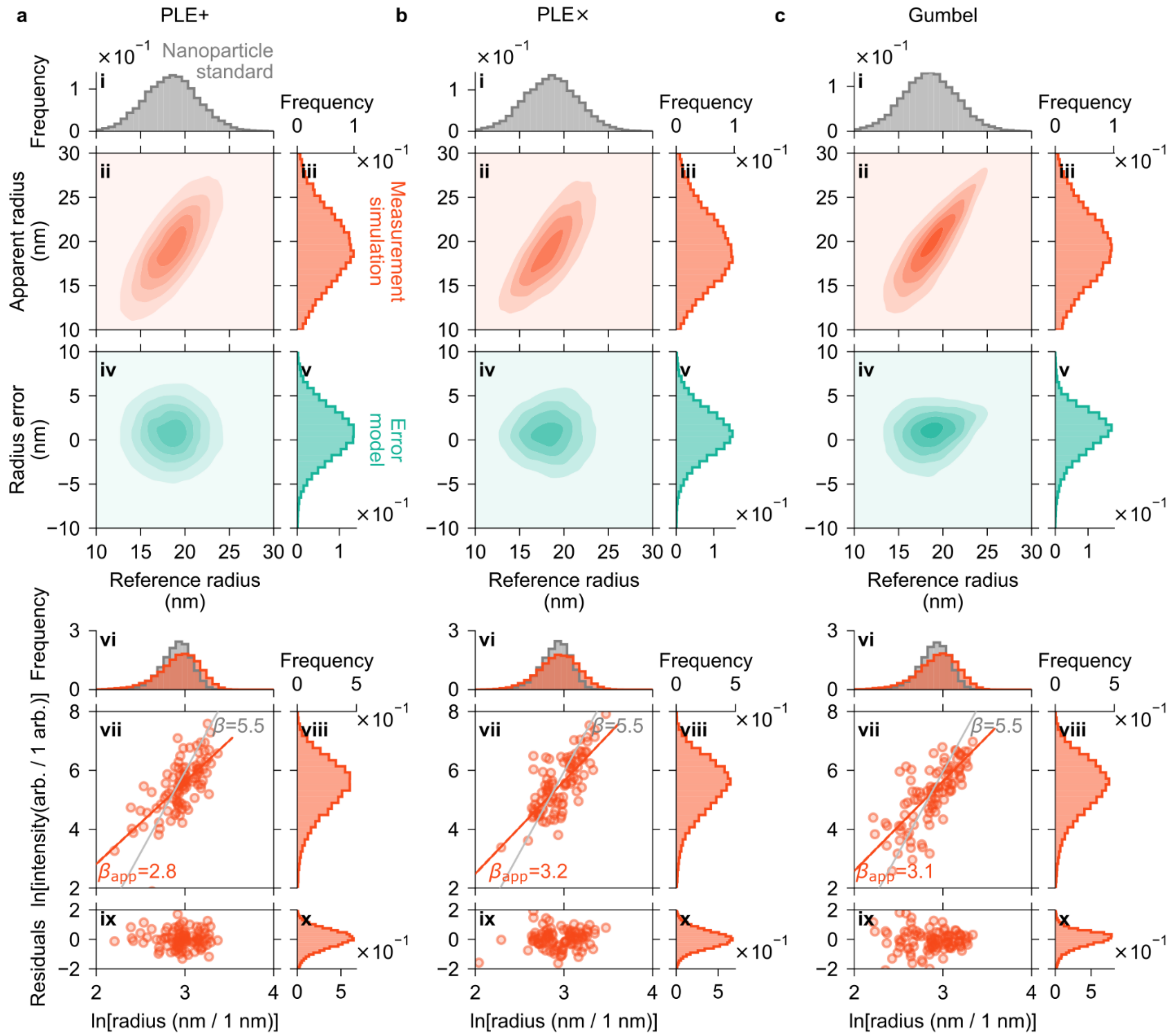

Figure S11. Corrections for Figure 2 of Reference 13. (a) PLE+, (b) PLE×, and (c) Gumbel models of errors of diameter apparent in Figure 2 of ref 13. (i–iii) Marginal and joint histograms showing the correlation of reference and apparent radii. The joint histogram in (c, ii) results from sampling the bivariate Gumbel copula with a correlation parameter of $\theta = 2.5$. (iv–v) Joint and marginal histogram showing correlation of reference radius and radius errors. (vi–x) Marginal histograms and scatter plots showing a bias that is evident as attenuation of an exponent or equivalent slope of a linear model of ln[intensity (arb. / 1 arb.)] versus ln[radius (nm / 1 nm)]. (vi) Marginal histograms showing (solid gray) ln[radius (nm / 1 nm)] data without measurement errors and (orange) apparent ln[radius (nm / 1 nm)] data approximating those of Figure 2 of ref 13. (vii) Scatter plot showing (orange circles) ln[intensity (arb. / 1 arb.)] versus ln[radius (nm / 1 nm)] data and (dark orange) least squares line and (gray) true line used for simulation. True exponents in (a, vii) and (b, vii) are approximately from eq 3, whereas the correction in (c, vii) is from manual selection to approximate the scatter in Figure 2 of ref 13. (viii) Marginal histogram showing intensity measurements. (ix) Scatter plot and (x) marginal histogram showing residual values of ln[intensity (arb. / 1 arb.)] from the least squares fit in (vii).

Note S6. Correction Errors due to Incorrect Assumptions

If reference data are unavailable, then an exponent correction is still possible on the basis of an evaluation of measurement uncertainty for the independent variable. Such a measurement error model involves assumptions that the mean value has zero bias and that the measurement uncertainty is the excess variability of the apparent values. Such assumptions can be overoptimistic,[31] and the exponent correction is sensitive to both an underestimate of measurement uncertainty and a bias of the mean value. We assess the resulting errors.

Assuming an estimate of the measurement uncertainty variance, $\sigma_{\mathrm{unc}}^2 = (1-k)\sigma_{\mathrm{true}}^2$, where $0 \leq k \leq 1$ and σ_{true}^2 is the true value of the measurement uncertainty variance, the relative error of the correction factor is

$$\frac{\dfrac{\sigma_{\mathrm{app}}^2}{\sigma_{\mathrm{app}}^2 - \sigma_{\mathrm{unc}}^2} - \dfrac{\sigma_{\mathrm{app}}^2}{\sigma_{\mathrm{app}}^2 - \sigma_{\mathrm{true}}^2}}{\dfrac{\sigma_{\mathrm{app}}^2}{\sigma_{\mathrm{app}}^2 - \sigma_{\mathrm{true}}^2}} = \frac{\left[\dfrac{\sigma_{\mathrm{app}}^2}{\sigma_{\mathrm{app}}^2 - \sigma_{\mathrm{unc}}^2}\right]}{\left[\dfrac{\sigma_{\mathrm{app}}^2}{\sigma_{\mathrm{app}}^2 - \sigma_{\mathrm{true}}^2}\right]} - 1 = \frac{\dfrac{1}{1 - \dfrac{(1-k)\sigma_{\mathrm{true}}^2}{\sigma_{\mathrm{app}}^2}}}{\dfrac{1}{1 - \dfrac{\sigma_{\mathrm{true}}^2}{\sigma_{\mathrm{app}}^2}}} - 1 = \frac{1 - \dfrac{\sigma_{\mathrm{true}}^2}{\sigma_{\mathrm{app}}^2}}{1 - \dfrac{(1-k)\sigma_{\mathrm{true}}^2}{\sigma_{\mathrm{app}}^2}} - 1 \quad \text{(S14)}$$

If $k = 0.2$, for example, then σ_{unc}^2 is too small by 20%, or, in terms of the standard deviation rather than the variance, σ_{unc} is too small by approximately 10%. If $\sigma_{\mathrm{true}}^2/\sigma_{\mathrm{app}}^2 = 0.20$, such that σ_{app}^2 is too large by 20%, or σ_{app} is too large by approximately 10%, then the correction factor is too small by approximately 5%.

The assumption of zero bias corresponds to $\mu_{\mathrm{a}} = 0$ for PLE+ and $\mu_{\mathrm{m}} = 1$ for PLE×. Upon violation of these assumptions, the relative error of the correction factors for PLE+ and PLE× are, respectively, eqs S15 and S16

$$\frac{\dfrac{\sigma_{\mathrm{ref}}^2 + \sigma_{\mathrm{a}}^2}{\sigma_{\mathrm{ref}}^2} - \dfrac{\sigma_{\mathrm{ref}}^2 + \sigma_{\mathrm{a}}^2}{\sigma_{\mathrm{ref}}^2}\left[1 + \dfrac{\mu_{\mathrm{a}}}{\mu_{\mathrm{ref}}}\right]^{-2}}{\dfrac{\sigma_{\mathrm{ref}}^2 + \sigma_{\mathrm{a}}^2}{\sigma_{\mathrm{ref}}^2}\left[1 + \dfrac{\mu_{\mathrm{a}}}{\mu_{\mathrm{ref}}}\right]^{-2}} = \frac{\mu_{\mathrm{a}}^2}{\mu_{\mathrm{ref}}^2} + \frac{2\mu_{\mathrm{a}}}{\mu_{\mathrm{ref}}} \quad \text{(S15)}$$

$$\frac{\dfrac{\dfrac{\sigma_{\mathrm{ref}}^2}{\mu_{\mathrm{ref}}^2} + \sigma_{\mathrm{m}}^2}{\dfrac{\sigma_{\mathrm{ref}}^2}{\mu_{\mathrm{ref}}^2}} - \dfrac{\dfrac{\sigma_{\mathrm{ref}}^2}{\mu_{\mathrm{ref}}^2} + \dfrac{\sigma_{\mathrm{m}}^2}{\mu_{\mathrm{m}}^2}}{\dfrac{\sigma_{\mathrm{ref}}^2}{\mu_{\mathrm{ref}}^2}}}{\dfrac{\dfrac{\sigma_{\mathrm{ref}}^2}{\mu_{\mathrm{ref}}^2} + \dfrac{\sigma_{\mathrm{m}}^2}{\mu_{\mathrm{m}}^2}}{\dfrac{\sigma_{\mathrm{ref}}^2}{\mu_{\mathrm{ref}}^2}}} = \frac{\sigma_{\mathrm{m}}^2\mu_{\mathrm{ref}}^2(\mu_{\mathrm{m}}^2 - 1)}{\sigma_{\mathrm{ref}}^2\mu_{\mathrm{m}}^2 + \sigma_{\mathrm{m}}^2\mu_{\mathrm{ref}}^2} \quad \text{(S16)}$$

From eq S15 for PLE+, for instance, an incorrect assumption of zero bias for an actual bias of $\mu_{\mathrm{a}}/\mu_{\mathrm{ref}} = \pm 2.5\%$ causes an error of the correction factor of approximately ±5%.

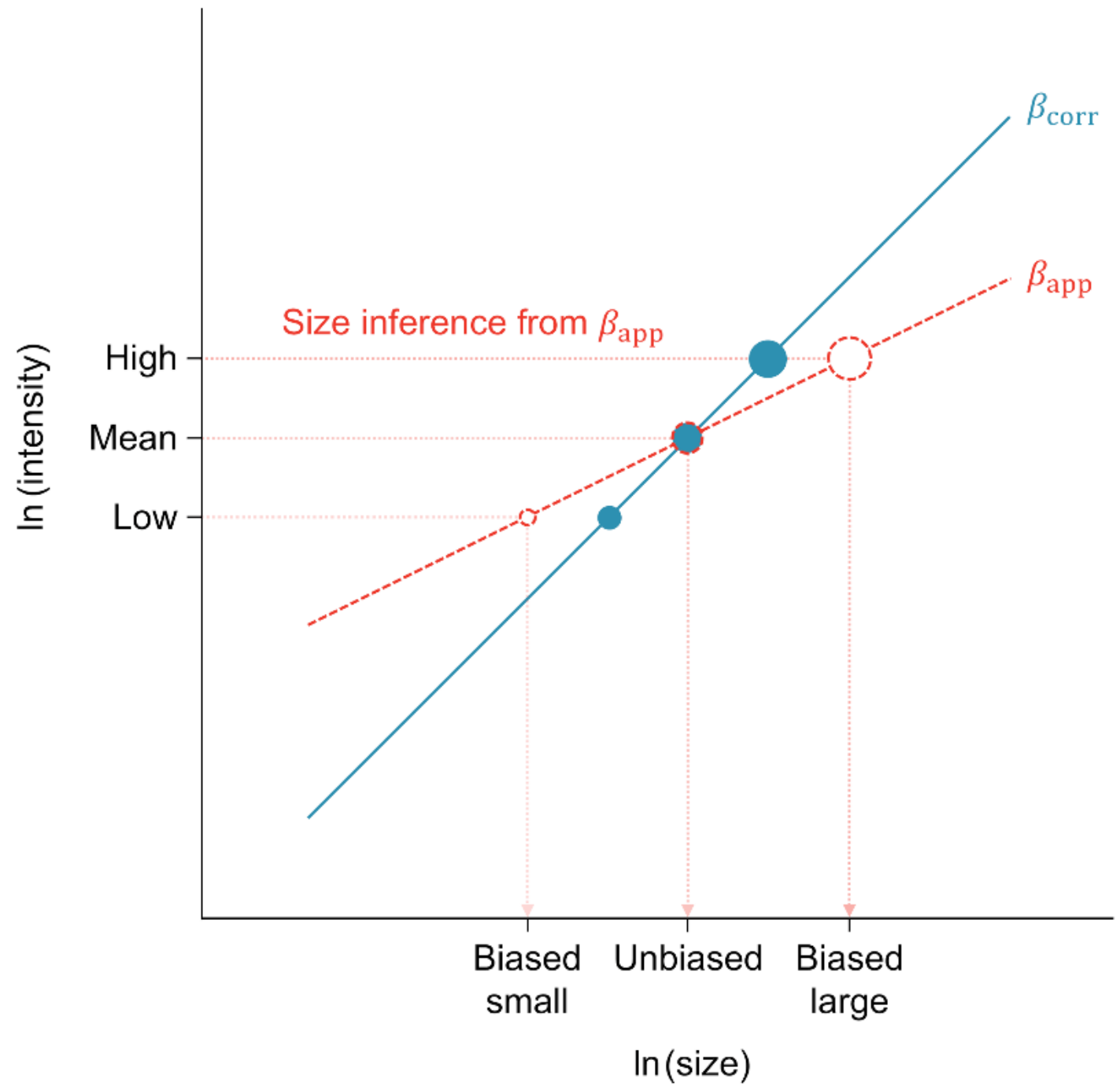

Figure S12. Size Inference from Apparent Exponent. Schematic diagram depicting a comparison of size inference results from apparent and corrected exponents.

REFERENCES

(1) Stavis, S. M.; Fagan, J. A.; Stopa, M.; Liddle, J. A. Nanoparticle Manufacturing – Heterogeneity through Processes to Products. *ACS Applied Nano Materials* **2018**, *1* (9), 4358-4385.

(2) García de Arquer, F. P.; Talapin, D. V.; Klimov, V. I.; Arakawa, Y.; Bayer, M.; Sargent, E. H. Semiconductor quantum dots: Technological progress and future challenges. *Science* **2021**, *373* (6555), eaaz8541.

(3) Kulkarni, S. A.; Mhaisalkar, S. G.; Mathews, N.; Boix, P. P. Perovskite Nanoparticles: Synthesis, Properties, and Novel Applications in Photovoltaics and LEDs. *Small Methods* **2019**, *3* (1), 1800231.

(4) Jin, R.; Zeng, C.; Zhou, M.; Chen, Y. Atomically Precise Colloidal Metal Nanoclusters and Nanoparticles: Fundamentals and Opportunities. *Chemical Reviews* **2016**, *116* (18), 10346-10413.

(5) Wang, N.; Thameem Dheen, S.; Fuh, J. Y. H.; Senthil Kumar, A. A review of multi-functional ceramic nanoparticles in 3D printed bone tissue engineering. *Bioprinting* **2021**, *23*, e00146.

(6) Xu, L.; Wang, X.; Liu, Y.; Yang, G.; Falconer, R. J.; Zhao, C.-X. Lipid Nanoparticles for Drug Delivery. *Advanced NanoBiomed Research* **2022**, *2* (2), 2100109.

(7) Lamoree, M. H.; van Boxel, J.; Nardella, F.; Houthuijs, K. J.; Brandsma, S. H.; Béen, F.; van Duursen, M. B. M. Health impacts of microplastic and nanoplastic exposure. *Nature Medicine* **2025**, *31* (9), 2873-2887.

(8) Tcherniak, A.; Ha, J. W.; Dominguez-Medina, S.; Slaughter, L. S.; Link, S. Probing a Century Old Prediction One Plasmonic Particle at a Time. *Nano Letters* **2010**, *10* (4), 1398-1404.

(9) Block, S.; Fast, B. J.; Lundgren, A.; Zhdanov, V. P.; Höök, F. Two-dimensional Flow Nanometry of Biological Nanoparticles for Accurate Determination of their Size and Emission Intensity. *Nature Communications* **2016**, *7* (1), 12956.

(10) Liao, K.-T.; Schumacher, J.; Lezec, H. J.; Stavis, S. M. Subnanometer Structure and Function From Ion Beams Through Complex Fluidics to Fluorescent Particles. *Lab on a Chip* **2018**, *18* (1), 139-152.

(11) Liao, K.-T.; Madison, A. C.; Pintar, A. L.; Ilic, B. R.; Copeland, C. R.; Stavis, S. M. A lateral nanoflow assay reveals surprising nanoplastic fluorescence. *arXiv* **2020**, *2101.03881*.

(12) Kamanzi, A.; Gu, Y.; Tahvildari, R.; Friedenberger, Z.; Zhu, X.; Berti, R.; Kurylowicz, M.; Witzigmann, D.; Kulkarni, J. A.; Leung, J.; Andersson, J.; Dhalin, A.; Höök, F.; Sutton, M.; Cullis, P. R.; Leslie, S. Simultaneous, Single-Particle Measurements of Size and Loading Give Insights into the Structure of Drug-Delivery Nanoparticles. *ACS Nano* **2021**, *15* (12), 19244-19255.

(13) Kamanzi, A.; Sutton, M.; Höök, F.; Leslie, S. Response to "Letter to the Editor Concerning 'Simultaneous, Single-Particle Measurements of Size and Loading Give Insights into the Structure of Drug-Delivery Nanoparticles'". *ACS Nano* **2023**, *17* (10), 8843-8846.

(14) Midtvedt, B.; Olsén, E.; Eklund, F.; Höök, F.; Adiels, C. B.; Volpe, G.; Midtvedt, D. Fast and Accurate Nanoparticle Characterization Using Deep-Learning-Enhanced Off-Axis Holography. *ACS Nano* **2021**, *15* (2), 2240-2250.

(15) Li, S.; Hu, Y.; Lin, J.; Schneiderman, Z.; Shao, F.; Wei, L.; Li, A.; Hsieh, K.; Kokkoli, E.; Curk, T.; Mao, H.-Q.; Wang, T.-H. Single-Particle Spectroscopic Chromatography Reveals Heterogeneous RNA Loading and Size Correlations in Lipid Nanoparticles. *ACS Nano* **2024**.

(16) Mohapatra, J.; Zeng, F.; Elkins, K.; Xing, M.; Ghimire, M.; Yoon, S.; Mishra, S. R.; Liu, J. P. Size-dependent magnetic and inductive heating properties of Fe_3O_4 nanoparticles: scaling laws across the superparamagnetic size. *Physical Chemistry Chemical Physics* **2018**, *20* (18), 12879-12887.

(17) Saint-Lager, M. C.; Laoufi, I.; Bailly, A.; Robach, O.; Garaudée, S.; Dolle, P. Catalytic properties of supported gold nanoparticles: new insights into the size-activity relationship gained from in operando measurements. *Faraday Discussions* **2011**, *152* (0), 253-265.

(18) Singhal, A.; Agur Sevink, G. J. The role of size and nature in nanoparticle binding to a model lung membrane: an atomistic study. *Nanoscale Advances* **2021**, *3* (23), 6635-6648.

(19) Mulholland, G. W.; Duelge, K. J.; Hackley, V. A.; Farkas, N.; Kramar, J. A.; Zachariah, M. R.; Takahata, K.; Sakurai, H.; Ehara, K. Measurement of 100 nm monodisperse particles by four Accurate methods: Traceability and uncertainty. *Aerosol Science and Technology* **2024**, *58* (3), 323-333.

(20) Guo, W. J.; Ma, S.; Wang, H.; Qiao, L.; Chen, L.; Hong, C.; Liu, B.; Zheng, X.; Peng, H. Q. Unveiling size-fluorescence correlation of organic nanoparticles and its use in nanoparticle size determination. *Aggregate* **2024**, *5* (1), e415.

(21) Liu, Z.; Sun, Z.; Di, W.; Qin, W.; Yuan, Z.; Wu, C. Brightness Calibrates Particle Size in Single Particle Fluorescence Imaging. *Optics Letters* **2015**, *40* (7), 1242-1245.

(22) Madison, A. C.; Pintar, A. L.; Copeland, C. R.; Farkas, N.; Stavis, S. M. Letter to the Editor Concerning "Simultaneous, Single-Particle Measurements of Size and Loading Give Insights into the Structure of Drug-Delivery Nanoparticles". *ACS Nano* **2023**, *17* (10), 8837-8842.

(23) Buonaccorsi, J. P. *Measurement Error: Models, Methods, and Applications*; CRC press, 2010.

(24) Carroll, R. J.; Ruppert, D.; Stefanski, L. A.; Crainiceanu, C. M. *Measurement Error in Nonlinear Models: A Modern Perspective*; Chapman and Hall/CRC, 2006.

(25) Fuller, W. A. *Measurement Error Models*; Wiley, 1987.

(26) Myers, R. H.; Montgomery, D. C.; Vining, G. G.; Robinson, T. J. *Generalized Linear Models: with Applications in Engineering and the Sciences*; John Wiley & Sons, 2012.

(27) Hausman, J. Mismeasured Variables in Econometric Analysis: Problems from the Right and Problems from the Left. *Journal of Economic perspectives* **2001**, *15* (4), 57-67.

(28) Bartel, T.; Stoudt, S.; Possolo, A. Force Calibration using Errors-in-Variables Regression and Monte Carlo Uncertainty Evaluation. *Metrologia* **2016**, *53* (3), 965.

(29) BIPM; IEC; IFCC; ILAC; ISO; IUPAC; IUPAP; OIML. *Evaluation of Measurement Data --- Guide to the Expression of Uncertainty in Measurement*; Joint Committee for Guides in Metrology, JCGM 100:2008, 2008. https://www.bipm.org/documents/20126/2071204/JCGM_100_2008_E.pdf/cb0ef43f-baa5-11cf-3f85-4dcd86f77bd6.

(30) Possolo, A.; Pintar, A. L. Plurality of Type A evaluations of uncertainty. *Metrologia* **2017**, *54* (5), 617.

(31) Thompson, M.; Ellison, S. L. R. Dark uncertainty. *Accreditation and Quality Assurance* **2011**, *16* (10), 483-487.

(32) Possolo, A. Simple Guide for Evaluating and Expressing the Uncertainty of NIST Measurement Results. In *NIST Technical Note 1900*, 2015.

(33) van Smeden, M.; Lash, T. L.; Groenwold, R. H. H. Reflection on modern methods: five myths about measurement error in epidemiological research. *International Journal of Epidemiology* **2019**, *49* (1), 338-347.

(34) Bellisario, D. O.; Ulissi, Z.; Strano, M. S. A Quantitative and Predictive Model of Electromigration-Induced Breakdown of Metal Nanowires. *The Journal of Physical Chemistry C* **2013**, *117* (23), 12373-12378.

(35) Kim, K. H.; Lara-Avila, S.; Kang, H.; He, H.; Eklöf, J.; Hong, S. J.; Park, M.; Moth-Poulsen, K.; Matsushita, S.; Akagi, K.; Kubatkin, S.; Park, Y. W. Apparent Power Law Scaling of Variable Range Hopping Conduction in Carbonized Polymer Nanofibers. *Scientific Reports* **2016**, *6* (1), 37783.
